# Associations of Total Legume, Pulse, and Soy Consumption with Incident Type 2 Diabetes: Federated Meta-Analysis of 27 Studies from Diverse World Regions

**DOI:** 10.1093/jn/nxaa447

**Published:** 2021-03-09

**Authors:** Matthew Pearce, Anouar Fanidi, Tom R P Bishop, Stephen J Sharp, Fumiaki Imamura, Stefan Dietrich, Tasnime Akbaraly, Maira Bes-Rastrollo, Joline W J Beulens, Liisa Byberg, Scheine Canhada, Maria del Carmen B Molina, Zhengming Chen, Adrian Cortes-Valencia, Huaidong Du, Bruce B Duncan, Tommi Härkänen, Maryam Hashemian, Jihye Kim, Mi Kyung Kim, Yeonjung Kim, Paul Knekt, Daan Kromhout, Camille Lassale, Ruy Lopez Ridaura, Dianna J Magliano, Reza Malekzadeh, Pedro Marques-Vidal, Miguel Ángel Martínez-González, Gráinne O'Donoghue, Donal O'Gorman, Jonathan E Shaw, Sabita S Soedamah-Muthu, Dalia Stern, Alicja Wolk, Hye Won Woo, EPIC-InterAct Consortium, Nicholas J Wareham, Nita G Forouhi

**Affiliations:** Medical Research Council Epidemiology Unit, University of Cambridge School of Clinical Medicine, CB2 0QQ, Cambridge, United Kingdom; Medical Research Council Epidemiology Unit, University of Cambridge School of Clinical Medicine, CB2 0QQ, Cambridge, United Kingdom; Medical Research Council Epidemiology Unit, University of Cambridge School of Clinical Medicine, CB2 0QQ, Cambridge, United Kingdom; Medical Research Council Epidemiology Unit, University of Cambridge School of Clinical Medicine, CB2 0QQ, Cambridge, United Kingdom; Medical Research Council Epidemiology Unit, University of Cambridge School of Clinical Medicine, CB2 0QQ, Cambridge, United Kingdom; Department of Molecular Epidemiology, German Institute of Human Nutrition, Nuthetal, Germany; Inserm U 1198, Montpellier University, Montpellier, France; Departments of Preventive Medicine and Public Health, University of Navarra, Pamplona, Spain; Spanish Biomedical Research Centre in Physiopathology of Obesity and Nutrition, Instituto de Salud Carlos III, Madrid, Spain; Navarra's Health Research Institute (IdiSNA), Pamplona, Spain; Department of Epidemiology & Biostatistics and the Amsterdam Public Health Institute, Amsterdam University Medical Center, HV, Amsterdam, The Netherlands; Department of Surgical Sciences, Uppsala University, Uppsala, Sweden; Faculty of Medicine, Federal University of Rio Grande do Sul, Porto Alegre, Brazil; Postgraduate Program in Nutrition and Health, Federal University of Espírito Santo, Vitória, Brazil; Medical Research Council Population Health Research Unit, University of Oxford, Oxford, United Kingdom; Clinical Trial Service Unit and Epidemiological Studies Unit, University of Oxford, Oxford, United Kingdom; Center for Research on Population Health, National Institute of Public Health, Cuernavaca, Mexico; Medical Research Council Population Health Research Unit, University of Oxford, Oxford, United Kingdom; Clinical Trial Service Unit and Epidemiological Studies Unit, University of Oxford, Oxford, United Kingdom; Faculty of Medicine, Federal University of Rio Grande do Sul, Porto Alegre, Brazil; Department of Public Health Solutions, Finnish Institute for Health and Welfare, Helsinki, Finland; Digestive Disease Research Center, Tehran University of Medical Sciences, Tehran, Iran; Biology Department, School of Arts and Sciences, Utica College, Utica, USA; Department of Preventive Medicine, Hanyang University, Seoul, South Korea; Department of Preventive Medicine, Hanyang University, Seoul, South Korea; National Research Institute of Health, Centers for Disease Control and Prevention, Cheongju, South Korea; Department of Public Health Solutions, Finnish Institute for Health and Welfare, Helsinki, Finland; Department of Epidemiology, University Medical Center Groningen, University of Groningen, Groningen, The Netherlands; Department of Epidemiology and Public Health, University College London, London, United Kingdom; Cardiovascular Risk and Nutrition Group, Hospital del Mar Research Institute (IMIM), Barcelona, Spain; Center for Research on Population Health, National Institute of Public Health, Cuernavaca, Mexico; Baker Heart and Diabetes Institute, Melbourne, Australia; Digestive Oncology Research Center, Tehran University of Medical Sciences, Tehran, Iran; Department of Medicine, Lausanne University Hospital, Lausanne, Switzerland; Departments of Preventive Medicine and Public Health, University of Navarra, Pamplona, Spain; Spanish Biomedical Research Centre in Physiopathology of Obesity and Nutrition, Instituto de Salud Carlos III, Madrid, Spain; Navarra's Health Research Institute (IdiSNA), Pamplona, Spain; Department of Nutrition, Harvard T.H. Chan School of Public Health, Boston, MA, USA; School of Public Health, Physiotherapy & Sports Science, University College Dublin, Dublin, Ireland; School of Health & Human Performance, Dublin City University, Dublin, Ireland; Baker Heart and Diabetes Institute, Melbourne, Australia; Center of Research on Psychological and Somatic Disorders (CoRPs), Tilburg University, Tilburg, The Netherlands; Institute for Food, Nutrition and Health, University of Reading, Reading, United Kingdom; National Council of Science and Technology (CONACyT)-Center for Research on Population Health, National Institute of Public Health, Cuernavaca, Mexico; Department of Surgical Sciences, Uppsala University, Uppsala, Sweden; Institute of Environmental Medicine, Karolinska Institutet, Stockholm, Sweden; Department of Preventive Medicine, Hanyang University, Seoul, South Korea; Medical Research Council Epidemiology Unit, University of Cambridge School of Clinical Medicine, CB2 0QQ, Cambridge, United Kingdom; Medical Research Council Epidemiology Unit, University of Cambridge School of Clinical Medicine, CB2 0QQ, Cambridge, United Kingdom

**Keywords:** legumes, diabetes, pulse, bean, peanut, lentil, pea, soy, chickpea

## Abstract

**Background:**

The consumption of legumes is promoted as part of a healthy diet in many countries but associations of total and types of legume consumption with type 2 diabetes (T2D) are not well established. Analyses across diverse populations are lacking despite the availability of unpublished legume consumption data in prospective cohort studies.

**Objective:**

To examine the prospective associations of total and types of legume intake with the risk of incident T2D.

**Methods:**

Meta-analyses of associations between total legume, pulse, and soy consumption and T2D were conducted using a federated approach without physical data-pooling. Prospective cohorts were included if legume exposure and T2D outcome data were available and the cohort investigators agreed to participate. We estimated incidence rate ratios (IRRs) and CIs of associations using individual participant data including ≤42,473 incident cases among 807,785 adults without diabetes in 27 cohorts across the Americas, Eastern Mediterranean, Europe, and Western Pacific. Random-effects meta-analysis was used to combine effect estimates and estimate heterogeneity.

**Results:**

Median total legume intake ranged from 0–140 g/d across cohorts. We observed a weak positive association between total legume consumption and T2D (IRR = 1.02, 95% CI: 1.01 to 1.04) per 20 g/d higher intake, with moderately high heterogeneity (*I*^2^ = 74%). Analysis by region showed no evidence of associations in the Americas, Eastern Mediterranean, and Western Pacific. The positive association in Europe (IRR = 1.05, 95% CI: 1.01 to 1.10, *I*^2^ = 82%) was mainly driven by studies from Germany, UK, and Sweden. No evidence of associations was observed for the consumption of pulses or soy.

**Conclusions:**

These findings suggest no evidence of an association of legume intakes with T2D in several world regions. The positive association observed in some European studies warrants further investigation relating to overall dietary contexts in which legumes are consumed, including accompanying foods which may be positively associated with T2D.

## Introduction

Type 2 diabetes (T2D) is a major worldwide public health issue which decreases both quality of life and life expectancy (1), with prevalence predicted to increase from 463 million adults in 2019 to 700 million by 2045 (2). Diet is a modifiable risk factor for T2D in addition to being a leading risk factor for overall mortality and morbidity worldwide (3). One dietary approach suggested to have potential benefit for T2D prevention is the consumption of legumes, owing to their low glycemic index (4) and high nutrient density characterized by high contents of dietary fiber, protein, B vitamins, and minerals (5). The consumption of legumes is also promoted in dietary recommendations, for example in the USA (6) and the UK (7), as well as the EAT-Lancet Commission (8). To date, the direction and strength of the association between legume consumption and T2D risk is not well established because of heterogeneity in published results (9–12). Studies in China (13) and Spain (14) reported inverse associations of total legume consumption with risk of T2D, but others found null associations in Australia and Europe (15–20), and a study from the USA suggested a positive association (21). Studies on types of legumes have mainly focused on soy products (13, 22–27) whereas evidence on pulses is sparse (13, 14).

Studying the association between legume consumption and T2D is complicated by inconsistency in the definition of legumes and legume subtypes. Legumes are defined as flowering plants in the Leguminosae botanical family, with the fruit enclosed in a pod; they include pulses such as beans, lentils, peas, and chickpeas (28), plus soybeans and peanuts (29). The consumption of legumes varies substantially across the world with people in some regions using legumes as staple foods (e.g. >200 g/d in some countries in South America, Central America, and Africa) and others having very low intakes (<10 g/d in parts of Eastern Europe and some Pacific Islands) (30). However, detailed investigations on such diversity in total legume consumption and its relation with incident T2D are lacking in diverse populations. Additionally, some cohorts have data on legume consumption, but have not reported the association with T2D, and as such our understanding may be limited by potential publication bias and regional differences. To address these concerns, this study used federated meta-analysis of harmonized individual-level data from 27 cohorts from different geographic locations. We aimed to examine the prospective association of total and types of legume intake with incident T2D in adults, and to investigate whether associations vary by population characteristics.

## Methods

### Cohorts and study variables

InterConnect aims to optimize the use of existing individual participant data by enabling cross-cohort analyses within consortia without the pooling of data at a central location. InterConnect uses a federated meta-analysis approach (31) and aims to build consortia within this infrastructure to answer specific research questions. We searched the InterConnect Data Discovery registry (http://www.interconnect-diabetes.eu/data-discovery/) to identify cohorts potentially suitable for inclusion in the legumes collaborative group. The InterConnect registry was compiled using systematic searches of the literature alongside surveys of other online study registries, surveys of websites relating to consortia of studies, and searches of the gray literature to identify unpublished data. We attempted to contact a total of 103 cohorts (see**Supplemental Table 1**), among which we were unable to establish contact with 46, a further 20 did not have sufficient data on exposure, outcome, or covariates, 4 stated they had no capacity to contribute, 4 declared no interest, and 2 cohorts had only recently started recruitment (see **Supplemental Figure 1**).

For the current research question on legumes, 27 prospective cohorts from diverse regions participated, and were included in the final collaborative group (see **Supplemental Table 2**). Data for 8 cohorts were obtained by approval of data sharing requests, whereas the remaining 19 cohorts uploaded data to a server to allow federated analysis. We classified regions according to the WHO (32), and included 7 cohorts from countries in the Americas (North and South America), 15 from countries in Europe, 4 from countries in the Western Pacific (Australia, China, Republic of Korea), and 1 from the Eastern Mediterranean (Iran). The Swedish Mammography Cohort and the Cohort of Swedish Men used the same protocol and were made available as 1 combined dataset. All cohorts obtained ethical review board approval at the host institution and written informed consent from participants.

### Dietary assessment

Of the 27 cohorts, 17 (63% of cohorts) used semiquantitative FFQs, 4 (15%) cohorts used a quantitative dietary questionnaire, 3 (11%) cohorts used an interviewer-administered dietary history, 2 (7%) cohorts used a 24-h recall, and 1 (4%) cohort used either an FFQ or a quantitative dietary questionnaire depending on location (see **Supplemental Table 3**). The majority of cohorts provided exposure data in the format of grams per day. For those that did not, variable-specific standard portion sizes sourced from the USDA (33) were used to convert frequency data to grams per day (see **Supplemental Table 4**).

Based on this definition, we used consumption in grams/day of the following food items: pulses intake, defined as the consumption of sum of pea, bean, chickpea, and lentil intakes; soy intake and total legume intake, defined as the consumption of sum of pulses, soy, and peanuts. We expressed observed associations per 20 g/d, approximating the median of total legume consumption across all the included cohorts, and being equivalent to 2 servings of 70 g per week or 140 g of legumes per week.

In some datasets, the exposure variables were prederived by the cohort, whereas in others the exposure variable was calculated by summing the consumption of separately reported foods. These summed variables were set to missing if any of the constituent food variables had missing values. For example, if data were missing for lentils but available (not missing) for peas, then the pulse variable would be set to missing for that participant as pulse includes both lentils and peas. However, if >10% of participants in a cohort had missing values for a constituent food variable, we consulted the host institution to ascertain whether data were truly missing or should be set to zero values. If all constituent food variables were missing, then the summed variable was set to missing.

### Incident T2D ascertainment

To minimize heterogeneity resulting from variation in T2D diagnosis across cohorts, we created 2 harmonized outcomes: the primary outcome was defined as “clinically incident T2D” and the secondary outcome was defined as “incident T2D.” For the primary outcome, a confirmed clinical case of incident T2D was considered as fulfilling any 1 or more of the following criteria: *1*) ascertained by linkage to a registry or medical record; *2*) confirmed antidiabetic medication usage; *3*) self-report of physician diagnosis or antidiabetic medication, verified by any of the following: a) ≥1 additional source from 1 or 2 above, b) biochemical measurement (glucose or glycated hemoglobin), c) a validation study with high concordance. For the secondary outcome, which was more inclusive, a case of incident T2D was confirmed by any of the following criteria: *1*) ascertained by linkage to a registry or medical record; *2*) confirmed antidiabetic medication usage; *3*) self-report of physician diagnosis or antidiabetic medication; or *4*) biochemical measurement (glucose or glycated hemoglobin).

### Statistical analyses

Federated analyses were conducted using R (R Core Team) within the DataSHIELD federated meta-analysis programming library. DataSHIELD permits analyses to be undertaken without the necessity for individual participant data to be transferred and stored at a central location (31). Instead, analyses are performed centrally with data remaining within the governance structure of the original cohort study.

For the main analyses, we excluded participants with a diagnosis of diabetes at baseline, those reporting extreme energy intakes (<500 or >3500 kcal/d for women and <800 or >4200 kcal/d for men) (34), and those with missing values for any of the exposure and outcome variables, as well as for the following potential confounding factors: age, sex, education, smoking, physical activity, alcohol intake, BMI, comorbidities (cancer, stroke, hypertension, myocardial infarction), and dietary covariates including the consumption of red and processed meat, fruit, vegetables, sugary beverages, dairy products, fish, and total energy. Covariates were not available in some cohorts (see **Supplemental Tables 5** and **6**).

Incidence rate ratios (IRRs) and 95% CI for T2D according to total and types of legume intake were estimated in each individual cohort using piecewise Poisson regression, which is available in the DataShield R programming library as a close approximation of the Cox model (35). For analyses using the European Prospective Investigation into Cancer (EPIC)–InterAct Study we applied a correction that is analogous to Prentice weighting (weights of 1 for all cases and weights of $\frac{{{\rm{\# noncases}}\,{\rm{in}}\,{\rm{whole}}\,{\rm{cohort}}}}{{{\rm{\# noncases}}\,{\rm{in}}\,{\rm{subcohort}}}}$ for noncases) for case cohort studies in survival analyses when using the piecewise Poisson method (36). Random-effects meta-analysis was used to combine effect estimates and to estimate the degree of heterogeneity (*I*^2^ statistic) using STATA/SE 14.2 (StataCorp).

To assess whether results varied by adjusting for different sets of covariates (see **Supplemental Table 7**), we fitted 4 models: Model 1 adjusted for sociodemographic characteristics and lifestyle behavioral covariates (age, sex, education, smoking, physical activity, alcohol, and energy intake); Model 2 additionally included BMI; Model 3 was as Model 2 plus prevalent baseline comorbidities (hypertension, cancer, stroke, and myocardial infarction); Model 4 was as Model 3 plus the consumption of red and processed meat, fruit, vegetables, sugary beverages, dairy products, and fish. Due to limited data on family history of diabetes and waist circumference, we conducted additional analyses fitting those covariates in the subgroup of participants for whom the information was available.

Tests for multiplicative interaction were performed in Model 4 for each of sex, age, and categories of BMI (<25 kg/m^2^, 25–30, >30) by adding a product term between legume intake and each of these variables separately to the regression analyses, with subsequent stratification if the *P*-interaction <0.05. Pooled effect sizes and *I*^2^ statistics (if applicable) were also presented for each geographic region represented (Americas, Eastern Mediterranean, Europe, and Western Pacific). To assess sources of heterogeneity between cohorts, meta-regression (if appropriate) was performed by regressing effect estimates on median intakes of legumes, geographic region, and dietary assessment method.

In the prespecified primary analyses, we observed a positive association between total legume intake and T2D incidence, and therefore we conducted post hoc exploratory analyses. We further assessed potential residual confounding due to the consumption of tea, coffee, cereal products, eggs, potato, soups, and sugars, as well as use of hormone replacement therapy (women only). This was conducted only in EPIC-InterAct due to the availability of additional data and the presence of outlying effect sizes. We also examined the potential for reverse causality by estimating associations with exclusion of T2D cases occurring in the first 2 y of follow-up. Due to large numbers of participants not consuming any legumes (74,440), we also estimated IRR in only those reporting total legume consumption >0 g/d. We investigated whether the high degree of heterogeneity in Europe was explained by effect sizes in Whitehall II, EPIC-InterAct Sweden, and EPIC-InterAct Germany by repeating the primary analysis with the omission of these studies.

## Results

The collaborative group of 27 cohorts included a total of 807,785 individuals (**Table 1**). Of the 27 cohorts included, 26 had not previously published on associations between legume consumption and T2D. Most participants were from the Western Pacific region (62%), followed by the Americas (22%), Europe (14%), and Eastern Mediterranean (1%). Two cohorts included only men, 3 included only women, and of the remainder, the percentage of women ranged from 29% to 83%. Median total legume intake ranged from 0 to 140 g/d across individual cohorts, tending to be higher in the Americas (mainly Latin America) and Asia than in Europe. Median soy consumption was zero except in China (34 g/d) and the Republic of Korea (22 and 39 g/d). In Europe and the Americas, legume consumption was mostly in the form of pulses. During follow-up periods ranging from 3.8 to 25.0 y a total of 36,750 clinically incident T2D cases were recorded as the primary outcome, and this number was 42,473 for incident T2D cases (secondary outcome).

**TABLE 1:** Baseline characteristics of participants in 27 cohorts to study the association between legume consumption and incident type 2 diabetes in InterConnect

	Analytical sample	Women	Age^2^	BMI^2^	New primary/secondary type 2 diabetes cases	Follow-up time^3^	Total legume intake^3^	Pulse intake^3^	Soy intake^3^
Study (country)^1^	*n*	%	y	kg/m^2^	*n*	y	g/day	g/day	g/day
Americas									
ARIC (USA)	9650	56	53.8 ± 5.7	27.2 ± 5.0	723/2005	9.5 (8.8, 23.2)	36 (23, 58)	33 (19, 51)	—
CARDIA (USA)	3920	59	25.0 ± 3.6	24.4 ± 4.8	198/198	25.0 (19.0, 25.0)	16 (4, 38)	2 (0, 9)	—
ELSA-Brasil (Brazil)	11,420	57	51.7 ± 9.0	26.7 ± 4.6	340/1009	3.8 (3.5, 4.1)	140 (67,280)	140 (67,280)	—
MESA (USA)	4922	54	61.7 ± 10.3	28.0 ± 5.2	228/692	9.0 (6.6, 10.0)	31 (15, 59)	20 (9, 39)	0 (0, 2)
MTC (Mexico)	59,829	100	41.8 ± 7.7	27.2 ± 4.7	1537/1674	6.5 (6.5, 6.5)	54 (26, 99)	45 (18, 80)	0 (0, 0)
PRHHP (Puerto Rico)	6977	0	54.1 ± 6.6	25.0 ± 3.9	310/825	5.0 (5.0, 5.0)	84 (0, 126)	84 (0, 126)	—
WHI (USA)	83,435	100	63.6 ± 7.4	26.5 ± 5.5	7728/7728	12.1 (8.8, 13.8)	28 (14, 51)	22 (11, 42)	0 (0, 0)
Eastern Mediterranean									
Golestan (Iran)	10,181	52	51.3 ± 7.9	26.8 ± 5.3	550/1191	4.1 (3.4, 5.6)	13 (8, 20)	12 (7, 18)	0 (0, 1)
Europe									
CoLaus (Switzerland)	3813	55	57.1 ± 10.4	25.8 ± 4.3	212/269	5.4 (5.3, 5.6)	3 (0, 8)	2 (0, 5)	0 (0, 0)
COSM/SMC (Sweden)	49,005	46	59.6 ± 8.8	25.2 ± 3.5	4780/4858	18.0 (18.0, 18.0)	28 (20, 52)	25 (20, 35)	0 (0, 16)
ELSA (UK)	7159	56	63.8 ± 9.4	28.0 ± 5.1	0/422	7.8 (5.8, 8.1)	0 (0, 0)	—	—
EPIC-InterAct France	795	100	56.9 ± 6.5	24.6 ± 4.7	257/257	9.3 (7.3, 10.5)	13 (3, 26)	—	—
EPIC-InterAct Germany	3448	50	52.4 ± 8.3	27.6 ± 4.8	1505/1505	9.5 (4.9, 11.2)	3 (1, 6)	—	—
EPIC-InterAct Italy	3112	65	51.4 ± 7.7	27.4 ± 4.8	1271/1271	10.9 (6.8, 12.7)	5 (2, 12)	—	—
EPIC-InterAct The Netherlands	2067	83	54.2 ± 10.0	26.6 ± 4.5	741/741	11.1 (6.4, 12.6)	7 (3, 14)	—	0 (0, 1)
EPIC-InterAct Spain	5584	57	50.4 ± 7.8	29.3 ± 4.6	2354/2354	12.5 (9.0, 13.6)	45 (26, 73)	—	—
EPIC-InterAct Sweden	5192	53	54.9 ± 9.7	26.7 ± 4.6	2383/2383	11.9 (9.2, 13.6)	0 (0, 1)	—	—
EPIC-InterAct UK	1858	53	58.3 ± 10.5	26.9 ± 4.5	608/608	10.6 (6.3, 12.2)	11 (6, 21)	—	0 (0, 0)
FMC (Finland)	9057	49	39.0 ± 15.6	24.8 ± 4.1	481/481	24.3 (22.5, 25.8)	5 (2, 8)	5 (2, 8)	—
Hoorn (The Netherlands)	1232	54	60.0 ± 6.7	26.2 ± 3.1	16/94	6.4 (6.1, 6.7)	11 (5, 22)	—	0 (0, 0)
SUN (Spain)	19,261	60	37.6 ± 12.0	23.5 ± 3.5	142/142	10.1 (5.9, 12.6)	21 (13, 30)	21 (13, 30)	—
Whitehall II (UK)	3991	29	49.6 ± 5.9	25.2 ± 3.7	466/586	16.2 (15.4, 16.6)	19 (10, 33)	16 (10, 25)	0 (0, 0)
Zutphen Elderly (The Netherlands)	475	0	71.0 ± 4.7	25.7 ± 2.9	11/61	10.2 (5.3, 10.3)	0 (0, 14)	0 (0, 16)	0 (0, 0)
Western Pacific									
AusDiab (Australia)	6153	52	51.3 ± 7.9	26.8 ± 5.3	196/387	11.7 (5.1, 12.2)	26 (16, 40)	25 (15, 38)	0 (0, 1)
CKB (China)	482,589	59	51.1 ± 10.6	23.6 ± 3.3	9601/9601	7.2 (6.3, 8.1)	34 (9, 34)	—	34 (9, 34)
KoGES A&A (Republic of Korea)	5279	52	50.6 ± 8.6	24.5 ± 3.0	81/790	7.7 (3.8, 7.8)	42 (25, 68)	2 (1, 3)	39 (23, 64)
KoGES CAVAS (Republic of Korea)	7381	64	61.5 ± 9.9	24.2 ± 3.1	31/341	4.1 (3.1, 5.5)	23 (11, 47)	0 (0, 1)	22 (10, 44)

1 ARIC, Atherosclerosis Risk in Communities study; AusDiab, the Australian Diabetes, Obesity and Lifestyle Study; CARDIA, the Coronary Artery Risk Development in Young Adults Study; CKB, the China Kadoorie Biobank; CoLaus, the Cohorte Lausannoise; COSM, the Cohort of Swedish Men; ELSA, the English Longitudinal Study of Ageing; ELSA-Brasil, the Brazilian Longitudinal Study of Adult Health; EPIC, the European Prospective Investigation into Cancer; FMC, the Finnish Mobile Clinic Health Examination Survey; KoGES CAVAS, Korean Genome and Epidemiology Study of Cardiovascular Disease Association; KoGES A&A, Korean Genome and Epidemiology Study Ansan and Ansung; MESA, the Multi-Ethnic Study of Atherosclerosis; MTC, the Mexican Teachers Cohort; PRHHP, the Puerto Rico Heart Health Program; SMC, the Swedish Mammography Cohort; SUN, the University of Navarra Follow-up Study; WHI, the Women's Health Initiative.

2 Values are mean  ± SD.

3 Values are median (IQR).

**FIGURE 1:**
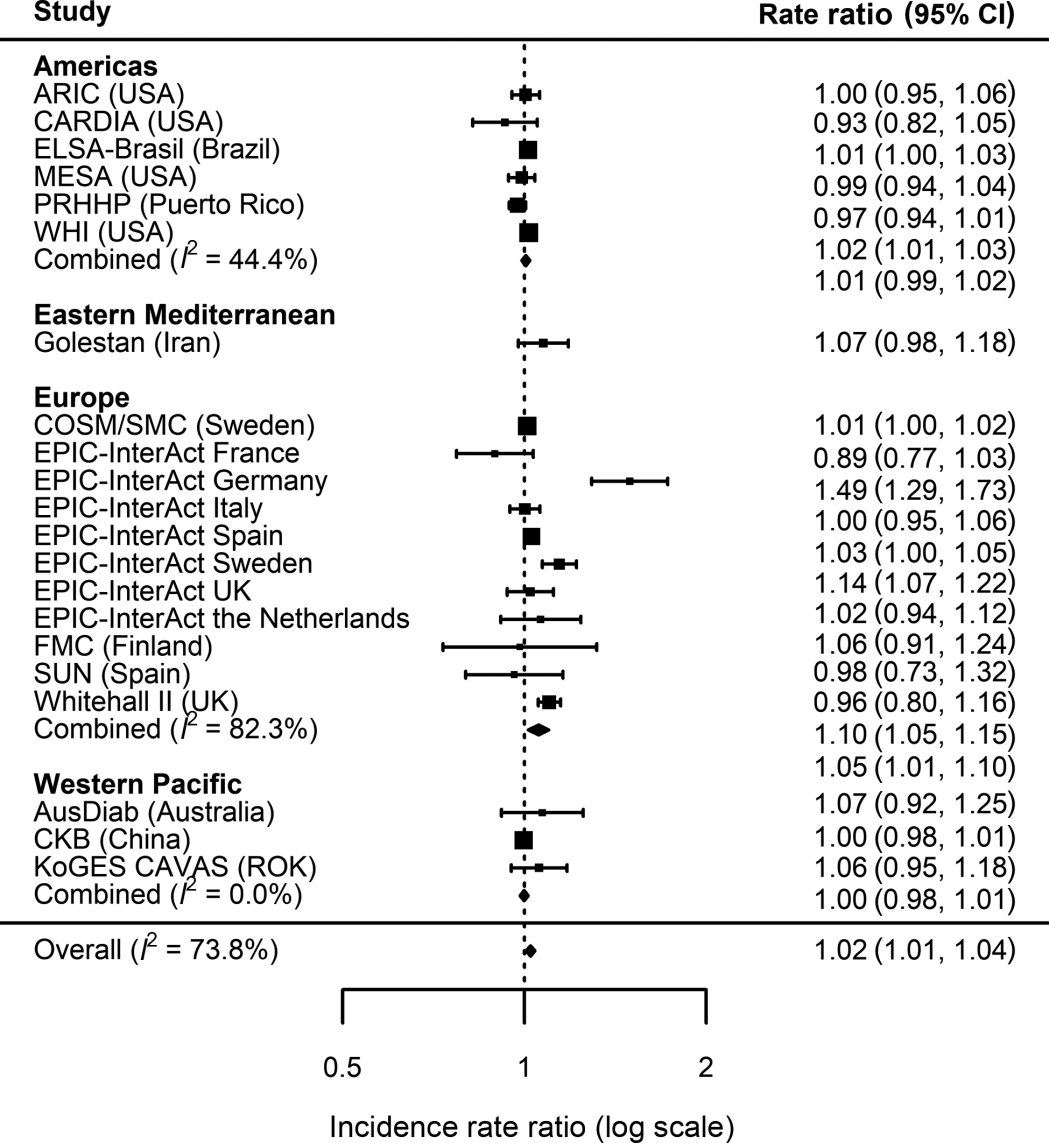
Incidence rate ratios and 95% CIs for the association between the consumption of total legumes (per 20 g/d) and incident type 2 diabetes (primary outcome) in InterConnect. Associations are adjusted for age, sex, education, smoking, physical activity, alcohol intake, total energy intake, BMI, comorbidity (hypertension, stroke, cancer, myocardial infarction) and other food intakes including fruit, vegetable, fish, red and processed meat, sugary drinks, and dairy products. Combined *n* = 729,998; total incident type 2 diabetes cases = 34,893. ARIC, Atherosclerosis Risk in Communities study; AusDiab, the Australian Diabetes, Obesity and Lifestyle Study; CARDIA, the Coronary Artery Risk Development in Young Adults Study; CKB, the China Kadoorie Biobank; COSM, the Cohort of Swedish Men; ELSA-Brasil, the Brazilian Longitudinal Study of Adult Health; EPIC, the European Prospective Investigation into Cancer; FMC, the Finnish Mobile Clinic Health Examination Survey; KoGES CAVAS, Korean Genome and Epidemiology Study of Cardiovascular Disease Association; MESA, the Multi-Ethnic Study of Atherosclerosis; PRHHP, the Puerto Rico Heart Health Program; ROK, Republic of Korea; SMC, the Swedish Mammography Cohort; SUN, the University of Navarra Follow-up Study; WHI, the Women's Health Initiative.

**FIGURE 2:**
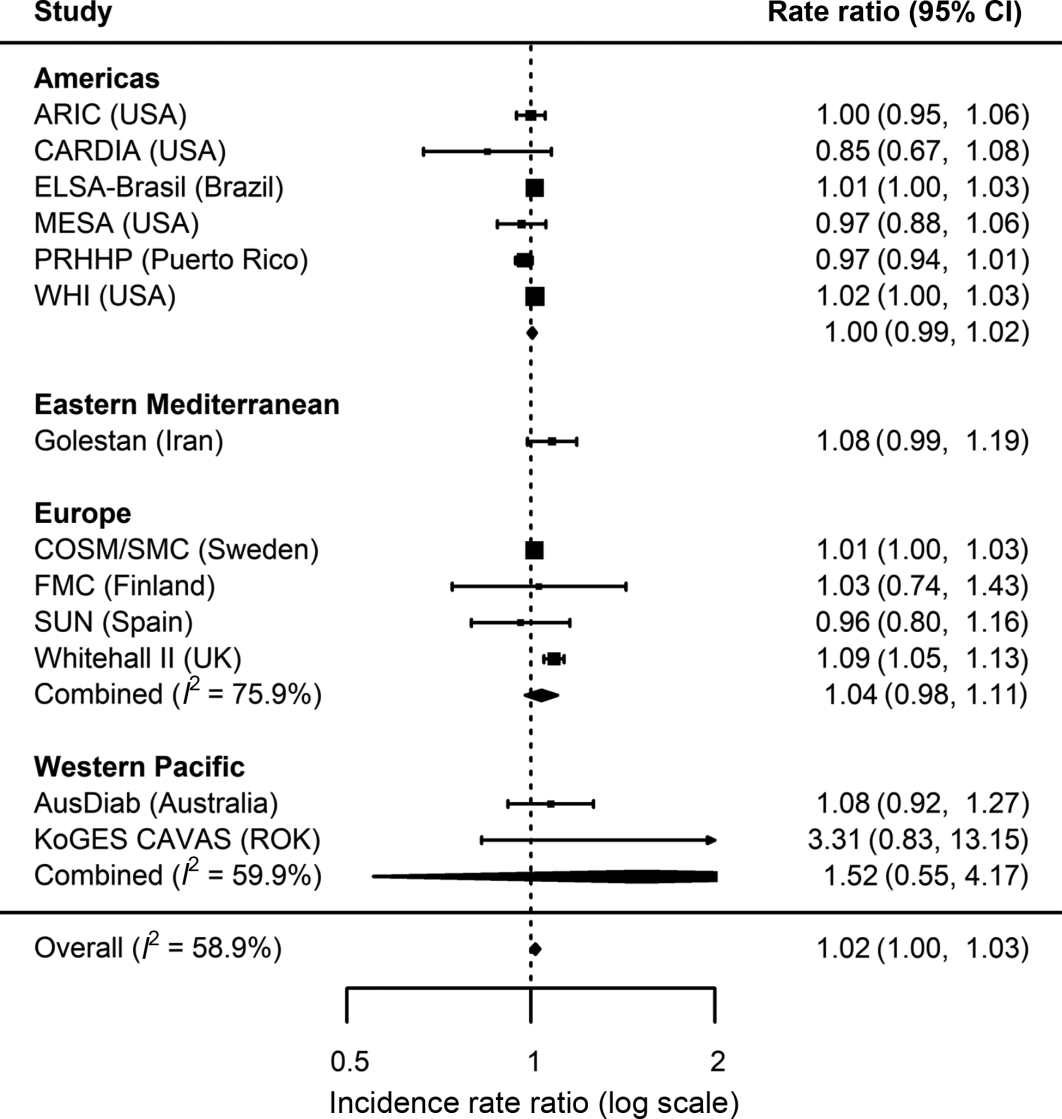
Incidence rate ratios and 95% CIs for the association between the consumption of pulses (per 20 g/d) and incident type 2 diabetes (primary outcome) in InterConnect. Associations are adjusted for age, sex, education, smoking, physical activity, alcohol intake, total energy intake, BMI, comorbidity (hypertension, stroke, cancer, myocardial infarction) and other food intakes including fruit, vegetable, fish, red and processed meat, sugary drinks, and dairy products. Combined *n* = 225,353; total incident type 2 diabetes cases = 16,173. ARIC, Atherosclerosis Risk in Communities study; AusDiab, the Australian Diabetes, Obesity and Lifestyle Study; CARDIA, the Coronary Artery Risk Development in Young Adults Study; COSM, the Cohort of Swedish Men; ELSA-Brasil, the Brazilian Longitudinal Study of Adult Health; EPIC, the European Prospective Investigation into Cancer; FMC, the Finnish Mobile Clinic Health Examination Survey; KoGES CAVAS, Korean Genome and Epidemiology Study of Cardiovascular Disease Association; MESA, the Multi-Ethnic Study of Atherosclerosis; PRHHP, the Puerto Rico Heart Health Program; ROK, Republic of Korea; SMC, the Swedish Mammography Cohort; SUN, the University of Navarra Follow-up Study; WHI, the Women's Health Initiative.

**FIGURE 3:**
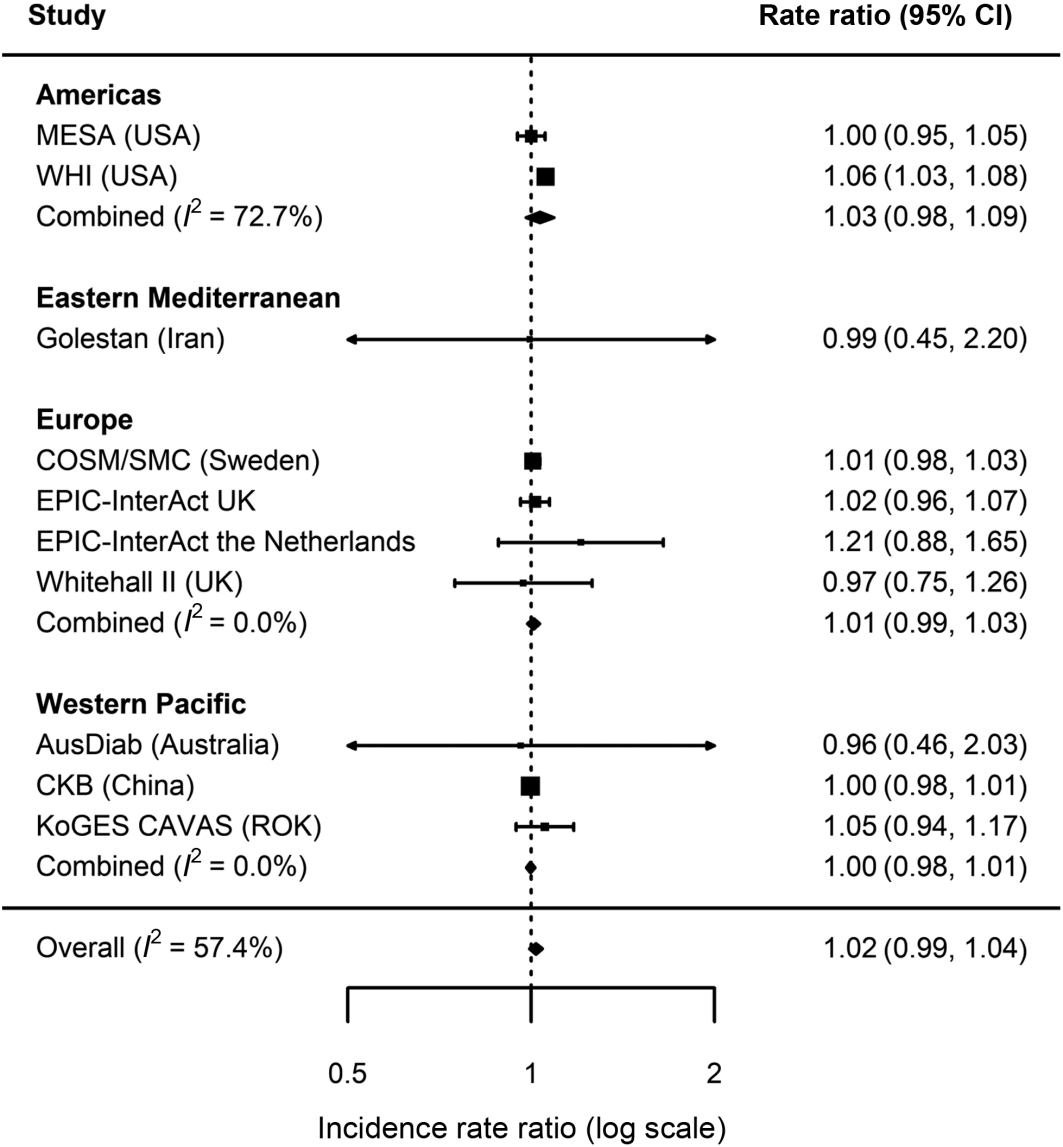
Incidence rate ratios and 95% CIs for the association between the consumption of soy products (per 20 g/d) and incident type 2 diabetes (primary outcome) in InterConnect. Associations are adjusted for age, sex, education, smoking, physical activity, alcohol intake, total energy intake, BMI, comorbidity (hypertension, stroke, cancer, myocardial infarction) and other food intakes including fruit, vegetable, fish, red and processed meat, sugary drinks, and dairy products. Combined *n* = 651,582; total incident type 2 diabetes cases = 24,929. AusDiab, the Australian Diabetes, Obesity and Lifestyle Study; CKB, the China Kadoorie Biobank; COSM, the Cohort of Swedish Men; EPIC, the European Prospective Investigation into Cancer; KoGES CAVAS, Korean Genome and Epidemiology Study of Cardiovascular Disease Association; MESA, the Multi-Ethnic Study of Atherosclerosis; ROK, Republic of Korea; SMC, the Swedish Mammography Cohort; WHI, the Women's Health Initiative.

Overall, there was a weak positive association between total legume consumption and T2D (IRR = 1.02, 95% CI: 1.01 to 1.04) per 20 g/d higher intake (equivalent to ∼2 servings/wk) (**Figure 1**) with moderately high heterogeneity (*I*^2^ = 74%). Analysis by region showed no evidence of associations in the Americas (IRR = 1.01, 95% CI: 0.99 to 1.02, *I*^2^ = 44%), Eastern Mediterranean (IRR = 1.07, 95% CI: 0.98 to 1.18; single study from Iran available), and Western Pacific (IRR = 1.00, 95% CI: 0.98 to 1.01, *I*^2^ = 0%). A positive association was observed in Europe where heterogeneity was high (IRR = 1.05, 95% CI: 1.01 to 1.10, *I*^2^ = 82%). The overall association for total legume corresponded to IRR = 1.17 (95% CI: 1.04 to 1.32) if scaled to 140 g/d higher consumption (equivalent to the largest median consumption for an individual cohort). No evidence for overall or region-specific associations was observed for pulses (**Figure 2**) or soy (**Figure 3**). Overall and region-specific results for total legumes, pulses, and soy were replicated when using the secondary outcome definition that included additional cohorts in the analysis (see **Supplemental Figures 2, 3**,and **4**).

There was no evidence of interactions of total or types of legume consumption with sex, age, or BMI (*P*-interaction >0.05). In the subset of cohorts for which we were able to additionally adjust for family history of diabetes and waist circumference (*n* = 729,998, clinically incident T2D cases = 34,893), there were only minor changes in the observed associations between total legume intake and incident T2D (see Supplemental Table 6). In meta-regression to examine potential sources of heterogeneity for the primary results, region (*P* value = 0.08), median consumption (*P* value = 0.12), and method of dietary assessment (*P* value = 0.29) did not appear to be strong predictors for the heterogeneity in effect size between cohorts.

To explore the possibility of reverse causality, we repeated the analysis excluding T2D events in the first 2 y of follow-up and observed a positive association between total legume consumption and T2D, similar to the primary analysis (see **Supplemental Figure 5**). A positive association was also observed when excluding participants reporting zero consumption of legumes thus restricting the analysis to consumers only (see **Supplemental Figure 6**). To investigate potential residual confounding, we entered additional covariates to the model in a subset of cohorts with available data. Further adjustment did not meaningfully alter individual or pooled effect sizes, including the observed positive associations between legume intake and T2D in the EPIC-InterAct studies for Germany and Sweden (see **Supplemental Figure 7**). Omitting Whitehall II, EPIC-InterAct Sweden, and EPIC-InterAct Germany from the primary analysis resulted in a reduction of the *I*^2^ value from 82% to 0% in Europe. The *I*^2^ value for the overall pooled result including all regions reduced from 74% to 17%.

## Discussion

In this large federated meta-analysis using individual participant data including the largest number of incident T2D cases assembled to date, we found no evidence of an association between total legume intake and T2D in several world regions (the Americas, Western Pacific, and Eastern Mediterranean), but there was a modest positive association in Europe. We found no evidence of associations of any of the types of legumes (pulses or soy) with T2D in any of the 4 world regions.

The current study builds upon a recent systematic review and meta-analysis of 7 published studies which reported no evidence of an association between total legume intake and T2D, with evidence of heterogeneity (*I*^2^ = 85%) (10). The present federated analysis showed that overall, there was a weak positive association and that heterogeneity was also relatively high (*I*^2^ = 74%). However, because we were able to include data from 27 studies across different world regions, including previously unpublished studies, we identified that a positive association was evident in Europe, whereas no association was observed in the other regions represented. Similarly, the lack of an association that we observed for total soy intake and T2D in the current analysis was consistent with the prior meta-analysis (10) although heterogeneity was lower in our analysis (*I*^2^ = 57%) than the prior meta-analysis (*I*^2^ = 91%).

Our finding of a positive association between legume consumption and T2D in Europe was in disagreement with previous null (17, 19, 20) or inverse (14) associations in European observational cohorts. In the present study, we observed high heterogeneity across European studies (*I*^2^ = 82%) and the association appeared to be driven by a small number of cohorts, specifically Whitehall II (UK), EPIC-InterAct Germany, and EPIC-InterAct Sweden. Previous analysis in the EPIC-Potsdam Study reported that legume intake was associated with a dietary pattern linked to greater risk of T2D, and the authors discussed that this may be because of legume consumption in mixed dishes (e.g. in stews) that include pork or beef (37). Our analyses adjusted for the consumption of red and processed meat as well as other potential dietary confounders, including sugar-sweetened beverages, as modeled in previous studies of individual cohorts. However, we cannot rule out residual confounding due to the co-consumption of other dietary items that may accompany legumes in some settings, for instance added sugar in canned baked beans as consumed in the UK or rice consumed alongside beans in Brazil and Asian countries. In the current study we were not able to control for methods of preparation (as these data were not available), and therefore confounding due to cooking methods or other ingredients consumed alongside legumes may have occurred in particular populations. Our findings suggest that dietary contexts of legume consumption are important and this may be a plausible explanation for the inconsistency of findings in the existing literature (9).

We observed no evidence of associations of total legume consumption with T2D risk in the American continent, consistent with previous findings (16, 18). In contrast, the Nurses’ Health Study reported evidence of a positive association when comparing highest and lowest quintiles (HR = 1.14, 95% CI: 1.03–1.25) and when using a continuous exposure (HR = 1.23, 95% CI: 0.97–1.56, per serving/d) (21). Our study is the first one to report on legume intake and T2D in Latin American countries, where consumption was highest and no associations were observed. Associations were heterogeneous across the Americas (*I*^2^ = 44%) emphasizing the need to better understand population-specific associations with T2D.

We have contributed new evidence in the Western Pacific region, additional to reports from 2 previous studies. Villegas et al. (13) reported an RR of 0.62 (95% CI: 0.51–0.74) comparing Chinese women in the highest and lowest quintiles, whereas the Melbourne Collaborative Cohort Study (15) reported no evidence of an association, which is in agreement with the present findings. These conflicting results may partly reflect differences in the extent of covariate adjustment; for example Villegas et al. (13) adjusted for vegetable intake but no other food groups, resulting in the potential for residual confounding towards an inverse association due to legumes being part of a healthy dietary pattern. There was no association observed in the Eastern Mediterranean region (with a study from Iran), and this finding is novel as we could not identify any prior reports.

In addition to dissimilarities between populations and methods of analysis, the inconsistency of associations in the present study and the literature as a whole could be explained by true variation in the consumption of specific legume types between studies and/or variation in how well this consumption was assessed. It is plausible that the positive associations observed in some cohorts may be explained by higher levels of consumption of legumes cooked and prepared using methods not practiced elsewhere. Alternatively, legume consumption in some populations may be part of wider dietary patterns or habits not fully controlled for in our analyses. Nonetheless, no single cohort showed a significant inverse association between T2D and the consumption of total legumes, pulses, or soy in the current analyses. Inconclusive results from the experimental literature offer little explanation for our findings. One meta-analysis of randomized controlled trials reported that higher legume consumption was favorably associated with fasting blood glucose, blood insulin, and insulin resistance. However, heterogeneity between the 11 studies was high (*I*^2^ = 75%), mean sample size was small (*n* = 23), and mean consumption (152 g/d) was higher than any cohort described here (38). Moreover, the authors of that meta-analysis acknowledged the short duration of the RCTs (mean of 6.7 wk), low quality of the included studies, and publication bias favoring small positive trials. It is arguable that legume consumption is important within the overall context of a healthful dietary pattern – such as in a “prudent” diet (39), the Alternate Healthy Eating Index (40), and Mediterranean diet (41). It is also important to consider the observed IRR in the context of the level of consumption of legumes in each population. For instance, in locations where legumes are staple foods (e.g. Brazil, Mexico, and Puerto Rico), no associations were observed, whereas, in contrast, the strongest positive IRR were observed in cohorts with low consumption levels (e.g. Germany, UK, and Sweden).

A key strength of this work was the analysis of individual participant data from 27 cohorts, contributing the largest number of confirmed incident T2D cases to date (*n* = 36,750) for an analysis of legume consumption and T2D. Previous studies included between 266 and 4529 incident T2D cases, whereas a meta-analysis reporting no overall association included 7 published studies with a total of 11,232 cases (10). The federated approach overcame constraints of the physical pooling of data due to governance or ethical and resource issues. We were able to assemble cohorts from diverse world regions with greater variation in amount and types of legume consumption, including 11 countries for which no evidence has been reported previously and the first analyses in Latin America and Eastern Mediterranean regions. By more comprehensively capturing heterogeneity in dietary patterns, as well as the nutrient profile, processing, and preparation of legumes, we extended prior conventional meta-analyses which may be biased by publication. Variation in population characteristics and analytical approaches may explain previous inconsistent results, so our findings across multiple regions with adjustment for the same covariates overcame that limitation. Use of individual participant data enabled us to harmonize exposure and outcome variables, improving the compatibility of data across cohorts and permitting analysis of total legume intake as well as pulses and soy products.

This study has a number of limitations. There was variation in both the portion sizes used by each cohort and those which we assigned at analysis. The accuracy of these portion sizes may vary according to the type of legumes in each cohort; for example, the legume content of bean soup may be exaggerated by the portion size assigned. Dietary assessment is complex and prone to measurement error, and legume consumption is particularly difficult to capture consistently across different populations because of the wide variety of legume types and subtypes, and how they are prepared, cooked, and consumed, which we were unable to examine. Specific validity of legume intake from dietary assessment instruments is generally limited in the published literature and was not available for the current research. Although we made considerable efforts to harmonize the exposure variables, heterogeneity resulting from dietary assessment cannot be ruled out, and it may be necessary to examine legume intake with greater specificity. We used diet data measured only at baseline but recognize that intraindividual variation over time might be present which may bias our findings in an unknown direction. However, most published studies have used solely baseline dietary data (10), and some studies that used repeated measures reported a positive association between legume intake and T2D (21). Our analyses were adjusted for a number of demographic, clinical, behavioral, and dietary confounding factors, but the risk of residual confounding is present, especially because covariates were unavailable in some cohorts, and the measurement quality of covariates may vary. We endeavored to include studies from as many world regions as possible, but were limited by the lack of studies identified from Eastern Europe, Africa, South Asia, Central America, and South America, reflecting an important research gap and highlighting the need for epidemiological research in these locations. This is especially important since in some of these regions legumes are consumed as staple foods. Future work focusing on population subgroups with different patterns of legume consumption and confounding factors is also warranted, for example in immigrant populations. Our analyses also assumed a linear association between exposure and outcome because it was not possible to order a pooled dataset. This precluded analyses using splines or quantiles, although a previous meta-analysis did not suggest a nonlinear association (9).

In summary, legume consumption appeared to have a null association with T2D in most of the world regions we investigated, including those where they are consumed as staple foods. The modest positive associations observed in some European cohorts require further investigation to understand the underlying reasons including cooking methods as well as accompanying foods and overall dietary patterns. Until findings from such further research are available, individuals and health professionals should continue to follow existing regional or other dietary guidelines.

## Supplementary Material

nxaa447_Supplemental_File

## Notes

Funding for the InterConnect project was provided by the European Union Seventh Framework Programme (FP7/2007-2013) under grant agreement no. 602068. Additionally, investigators acknowledge funding from the following agencies: NJW, NGF, FI, and MP acknowledge funding from the Medical Research Council Epidemiology Unit (MC_UU_12015/1 and MC_UU_12015/5); NJW and NGF acknowledge support from National Institute of Health Research (NIHR) Biomedical Research Centre Cambridge: Nutrition, Diet, and Lifestyle Research Theme (IS-BRC-1215-20014). TB acknowledges funding from EUCAN-Connect under the European Union's Horizon 2020 research and innovation programme (grant agreement no. 824989). MBR and MAMG acknowledge funding from the Spanish Government Instituto de Salud Carlos III and the European Regional Development Fund (FEDER) (PI17/01795). JK acknowledges funding from the Korea Centers for Disease Control and Prevention, Ministry for Health and Welfare, Republic of Korea (4845-301 and 4851-302), and the Collaborative Genome Program for Fostering New Post-Genome Industry of the National Research Foundation (NRF) funded by the Ministry of Science and ICT (NRF-2017M3C9A6047623). RM acknowledges funding from the Tehran University of Medical Sciences (grant number: 81/15), Cancer Research UK (grant number: C20/A5860), the Intramural Research Program of the U.S. National Cancer Institute, NIH, and the International Agency for Research on Cancer. LB acknowledges that the Cohort of Swedish Men (COSM) and the Swedish Mammography Cohort (SMC) are part of the Swedish Infrastructure for Medical Population-Based Life-Course and Environmental Research (SIMPLER), which receives funding from the Swedish Research Council (2017-00644). BD is an investigator of the Brazilian National Health Technology Assessment Institute and SC received a fellowship from Conselho Nacional de Desenvolvimento Científico e Tecnológico (CNPq), the Brazilian National Council for Scientific and Technological Development Fund (FEDER) (PI17/01795). The Zutphen Elderly Study was funded by The Netherlands Prevention Foundation, The Hague, The Netherlands and the National Institute of Aging, Bethesda, MD, USA. The funding sources did not participate in the design or conduct of the study; collection, management, analysis, or interpretation of the data; or preparation, review, or approval of the manuscript.

Author disclosures: The authors report no conflicts of interest.

Supplemental Tables 1–7 and Supplemental Figures 1–9 are available from the “Supplementary data” link in the online posting of the article and from the same link in the online table of contents at https://academic.oup.com/jn/.

Abbreviations used: EPIC, the European Prospective Investigation into Cancer; IRR, incidence rate ratio; T2D, type 2 diabetes.
