# Supplementary material for: Associations of Total Legume, Pulse, and Soy Consumption with Incident Type 2 Diabetes: Federated Meta-Analysis of 27 Studies from Diverse World Regions
Source: J Nutr. 2021 Mar 9;151(5):1231–40. doi: 10.1093/jn/nxaa447 (PMC8112771; doi:10.1093/jn/nxaa447)
Supplement: nxaa447_Supplemental_File [file nxaa447_supplemental_file.docx]

**Associations of total legume, pulse, and soy consumption with incident type 2 diabetes: federated meta-analysis of 27 studies from diverse world regions**

Pearce et al.

Online Supplemental Material

**Supplemental Table 1** Names and locations of 103 studies contacted regarding participation in InterConnect legume project^1,2^

| Study name | Location | Participated in present study |
| --- | --- | --- |
| Prospective Urban and Rural Epidemiological study | Low and middle income countries (n=25) |  |
| Sympathetic activity and Ambulatory Blood Pressure in Africans prospective cohort study | Africa |  |
| Australian Longitudinal Study on Women s Health | Australia |  |
| Blue Mountains Eye Study | Australia |  |
| Melbourne Collaborative Cohort Study | Australia |  |
| Australian Diabetes, Obesity and Lifestyle Study | Australia | Yes |
| Brazilian Longitudinal Study of Adult Health | Brazil | Yes |
| Alberta's Tomorrow Project | Canada |  |
| BC Generations Project | Canada |  |
| Sandy lake health and diabetes project | Canada |  |
| Adventist Health Study-2 | Canada, United States |  |
| China Kadoorie Biobank | China | Yes |
| Hong Kong Dietary Survey | China |  |
| Shanghai Men's Health Study | China |  |
| Shanghai Women's Health Study | China |  |
| China Health and Nutrition Survey | China |  |
| Nutrition and Health of Aging Population in China | China |  |
| Wuhan-Zhuhai cohort study and Dongfeng-Tongji cohort | China |  |
| Copenhagen City Heart Study | Denmark |  |
| Copenhagen General Population Study | Denmark |  |
| Danish General Suburban Population Study | Denmark |  |
| Inter99 Study | Denmark |  |
| Estonian Genome Center of University of Tartu | Estonia |  |
| Botnia Prospective Study | Finland |  |
| National FINRISK Study transferred to form new study: FinHealth Study | Finland |  |
| Finnish Mobile Clinic Health Examination Survey | Finland | Yes |
| Epidemiological Study on the Insulin Resistance Syndrome | France |  |
| Cooperative Health Research in the Region Augsburg | Germany |  |
| Study of Health in Pomerania | Germany |  |
| Golestan Cohort Study | Iran | Yes |
| Prospective Epidemiological Research Studies of the Iranian Adults | Iran |  |
| European Prospective Investigation into Cancer and Nutrition - Turin | Italy |  |
| Saku Diabetes Study | Japan |  |
| Japan Collaborative Cohort Study for Evaluation of Cancer | Japan |  |
| Japan Public Health Center-based Prospective Study | Japan |  |
| Suita Study | Japan |  |
| Mexican Health and Ageing Study | Mexico |  |
| Mexican Teachers’ Cohort study | Mexico | Yes |
| Doetinchem Cohort Study | Netherlands |  |
| Hoorn Study | Netherlands | Yes |
| Rotterdam Study | Netherlands |  |
| Netherlands Cohort Study | Netherlands |  |
| Zutphen Elderly Study | Netherlands | Yes |
| Nord-Trøndelag Health Study | Norway |  |
| Norwegian Women and Cancer Study | Norway |  |
| Puerto Rico Heart Health Program | Puerto Rico | Yes |
| Korean Genome and Epidemiology Study Consortium^3^ | Republic of Korea | Yes |
| Singapore Chinese Health Study | Singapore |  |
| Singapore Consortium of Cohort Studies | Singapore |  |
| Seguimiento Universidad de Navarra (University of Navarra follow-up) | Spain | Yes |
| Prevencion con Dieta Mediterranea study | Spain |  |
| Cohort Of Swedish Men | Sweden | Yes |
| Malmö Preventive Project | Sweden |  |
| Stockholm Diabetes Prevention Program | Sweden |  |
| Swedish Mammography Cohort | Sweden | Yes |
| Cohorte Lausannoise Study | Switzerland | Yes |
| Swiss Cohort Study on Air Pollution and Lung and Heart Diseases in Adults | Switzerland |  |
| Hertfordshire Cohort Study | United Kingdom |  |
| British Regional Heart Study | United Kingdom |  |
| British Women's Heart and Health Study | United Kingdom |  |
| English Longitudinal Study of Ageing | United Kingdom | Yes |
| European Prospective Investigation into Cancer and Nutrition - Norfolk | United Kingdom |  |
| European Prospective Investigation into Cancer and Nutrition - Oxford | United Kingdom |  |
| National Survey of Health and Development | United Kingdom |  |
| Southampton Women's Survey | United Kingdom |  |
| Wales Electronic Cohort for Children | United Kingdom |  |
| Whitehall II study | United Kingdom | Yes |
| Agricultural Health Study | United States |  |
| Atherosclerosis Risk in Communities Study | United States | Yes |
| Black Women's Health Study | United States |  |
| Boston Area Community Health Survey | United States |  |
| Cardiovascular Health Study | United States |  |
| CATHeterization GENetics | United States |  |
| Coronary Artery Risk Development in Young Adults | United States | Yes |
| Framingham Offspring Study | United States |  |
| Genetic Study of Atherosclerosis Risk in Families | United States |  |
| Health Professionals Follow-Up Study | United States |  |
| Health, Aging, and Body Composition | United States |  |
| Howard University Family Study | United States |  |
| Iowa Women's Health Study | United States |  |
| Jackson Heart Study | United States |  |
| Kuakini Honolulu Heart Program new name: Honolulu-Asia Aging Study | United States |  |
| Mexican-American Cohort Study | United States |  |
| Multiethnic Cohort Study | United States |  |
| National Institutes of Health-American Association of Retired Persons Diet and Health Study | United States |  |
| Nurses Health Study I | United States |  |
| Nurses Health Study II | United States |  |
| Rancho Bernardo Study | United States |  |
| Strong Heart Study | United States |  |
| BetaGene study: Genetic predisposition for B-cell dysfunction in Mexican-American families of probands with gestational diabetes | United States |  |
| Health and Retirement Study | United States |  |
| Healthy Aging in Neighborhoods of Diversity across the Life Span study | United States |  |
| Insulin Resistance Atherosclerosis (IRAS) family study | United States |  |
| Multi-Ethnic Study of Atherosclerosis | United States | Yes |
| Northern Manhattan Study | United States |  |
| San Antonio Heart Study | United States |  |
| Women's Health Initiative - SNP Health Associated Resource | United States |  |
| Women’s Genome Health Study part of WHS study | United States |  |
| Women's Health Initiative | United States | Yes |
| Women's Health Study | United States |  |
| Reasons for Geographic and Racial Differences in Stroke Study | United States |  |
| Southern Community Cohort Study | United States |  |

^1^Data from European Prospective Investigation into Cancer and Nutrition (EPIC)-InterAct were available were available through an agreement with the InterAct consortium. This included 8 additional studies from France, Germany, Italy, Netherlands, Spain, Sweden, UK.

^2^Recruiment flowchart provided in Supplemental Figure 1.

^3^Provided data for two cohorts: Korean Genome and Epidemiology Study of Cardiovascular Disease Association; Korean Genome and Epidemiology Study Ansan and Ansung.

**Supplemental Table 2** Characteristics of 27 cohorts to study the association between legume consumption and incident type 2 diabetes in InterConnect

| Study^2^ | Location | Participants | Recruitment time-frame | Baseline sample size |
| --- | --- | --- | --- | --- |
| **Americas** |  |  |  |  |
| ARIC (1) | USA | Ethnically-representative men and women in four communities | 1987-1989 | 15,792 |
| CARDIA (2) | USA | Black and white men and women aged 18-30 years selected to be representative of four cities | 1985-1986 | 5115 |
| ELSA-Brasil (3) | Brazil | Civil servants aged 35-74 years from five universities and one research institute | 2008-2010 | 15,105 |
| MESA (4) | USA | Ethnically diverse, population-based sample of men and women aged 45-84 years | 2000-2002 | 6814 |
| MTC (5) | Mexico | Women working as public school teachers residing in a culturally, geographically and economically diverse 12-state area | 2006-2008 | 115,314 |
| PRHHP (6) | Puerto Rico | Rural and urban men aged 45-64 years | 1965-1968 | 9824 |
| WHI (7) | USA | Postmenopausal women | 1994-1998 | 9676 |
| **Eastern Mediterranean** |  |  |  |  |
| Golestan (8) | Iran | Healthy men and women aged 40-75 years from urban (20%) and rural areas (80%), including Turkmen (74 %) and Non-Turkmen (26%) ethnicity | 2004-2008 | 50,045 |
| **Europe** |  |  |  |  |
| CoLaus (9) | Switzerland | Caucasian men and women aged 35-75 years living in Lausanne | 2003-2006 | 6188 |
| COSM/SMC^1^ (10) | Sweden | Men born aged 45-79 years living in Västmanland and Örebro counties. Women aged 39-76 living in Uppsala Västmanland counties | 1997, 1987-1990 | 112,557 |
| ELSA (11) | UK | Men and women aged ≥ 50 years drawn from the Health Survey for England | 2006-2009 | 11,050 |
| EPIC-InterAct (12) | France, Germany, Italy, the Netherlands, Spain, Sweden, UK | A case-cohort of all type 2 diabetes cases occurring in EPIC cohorts between 1991 and 2007 from 8 of the 10 EPIC countries and a subcohort of individuals randomly selected from those with available stored blood and buffy coat, stratified by centre | 1991-1998 | 10,433 (cases);  14,228 (subcohort) |
| FMC (13) | Finland | Men and women aged 15-99 from 27 communities of rural or semi urban dwellers or employees of a factory | 1966-1972 | 10,054 |
| Hoorn (14) | the Netherlands | Men and women aged 60-75 years living in the town of Hoorn | 1989-1991 | 2484 |
| SUN (15) | Spain | Men and women who are university graduates aged ≥ 20 years | 1999-present | 22,057 |
| Whitehall II (16) | UK | Men and women working in the British Civil Service aged 35-55 years | 1985-1988 | 10,308 |
| Zutphen Elderly (17) | the Netherlands | Men aged 65-82 years living in town of Zutphen | 1985 | 939 |
| **Western Pacific** | | | | |
| AusDiab (18) | Australia | Men and women aged ≥ 25 years from the six states plus the Northern Territory | 1999-2000 | 11,247 |
| CKB (19) | China | Men and women aged 30-79 years from five urban and five rural regions | 2004-2008 | 512,891 |
| KoGES A&A(20) | Republic of Korea | Men and women aged ≥ 40 years in national health examinee registry | 2001-2002 | 10,030 |
| KoGES CAVAS (20) | Republic of Korea | Men and women aged ≥ 40 years in national health examinee registry | 2005-2011 | 21,715 |

^1^Analysed as one combined cohort.

^2^ARIC, Atherosclerosis Risk in Communities study; AusDiab, the Australian Diabetes, Obesity and Lifestyle Study; CARDIA, the Coronary Artery Risk Development in Young Adults Study; CKB, the China Kadoorie Biobank; CoLaus, the Cohorte Lausannoise; COSM, the Cohort of Swedish Men; ELSA, the English Longitudinal Study of Ageing; ELSA-Brasil, the Brazilian Longitudinal Study of Adult Health; EPIC, the European Prospective Investigation into Cancer; FMC, the Finnish Mobile Clinic Health Examination Survey; KoGES CAVAS, Korean Genome and Epidemiology Study of Cardiovascular Disease Association; KoGES A&A, Korean Genome and Epidemiology Study Ansan and Ansung; MESA, the Multi-Ethnic Study of Atherosclerosis; MTC, the Mexican Teachers Cohort; PRHHP, the Puerto Rico Heart Health Program; SMC, the Swedish Mammography Cohort; SUN, the University of Navarra Follow-up Study; WHI, the Women’s Health Initiative.

**Supplemental Table 3** Details of exposure variables used to study the association between legume consumption and incident type 2 diabetes in InterConnect

| Study^1^ | Dietary assessment method | Units provided | Total legume | Pulses | Soy products |
| --- | --- | --- | --- | --- | --- |
| ARIC | Interviewer-administered FFQ | Frequency per day/week/month | Beans or lentils  String or green beans  Peas or lima beans  Peanut butter | Beans or lentils  String or green beans  Peas or lima beans | Not available |
| CARDIA | Interviewer-administered dietary history | Serving per day | Legumes  Peanuts | Cooked dried beans | Not available |
| ELSA-Brasil | Interviewer-administered online FFQ | Grams per day | Total legumes | Beans  Bean stew  Lentils, chickpeas | Not available |
| MESA | FFQ | Serving per day | Beans  Pea soup  Green beans  Refried beans  Soy milk  Stir fried tofu  Tofu dessert  Peanuts | Beans  Pea soup  Green beans  Refried beans | Soy milk  Stir fried tofu  Tofu dessert |
| MTC | FFQ | Frequency per day/week/month | Kidney bean  Lentil or chickpeas  Peas  Fava beans  Green beans (ejotes)  Peanuts natural  Peanuts | Kidney bean  Lentil or chickpeas  Peas  Fava beans  Green beans (ejotes) | Soy milk |
| PRHHP | 24-hour recall | Quarter cup per day | Peas or beans | Peas or beans | Not available |
| WHI | FFQ | Serving per day | Green or English peas  Bean soups  Refried beans  Green or string beans  All other beans  Soy  Peanuts | Green or English peas  Bean soups  Refried beans  Green or string beans  All other beans | Soy |
| Golestan | FFQ | Grams per day | Lima bean  Green bean  Green pea  White bean  Red bean  Pinto bean  Pea  Split pea  Lentil  Peanut  Soy | Lima bean  Green bean  Green pea  White bean  Red bean  Pinto bean  Pea  Split pea  Lentil | Soy |
| CoLaus | FFQ | Grams per day | Green peas  Tofu | Green peas | Tofu |
| COSM/  SMC | FFQ | Grams per day | Green peas  Pea soup, beans, lentils  Soy products | Green peas  Pea soup, beans, lentils | Soy products |
| ELSA | 24-hour recall | Tablespoon per day | Pulses | Pulses | Not available |
| EPIC-InterAct France | Quantitative questionnaire | Grams per day | Legumes | Not available | Not available |
| EPIC-InterAct Germany | Quantitative questionnaire | Grams per day | Legumes | Not available | Not available |
| EPIC-InterAct Italy | FFQ or  quantitative questionnaire | Grams per day | Legumes | Not available | Not available |
| EPIC-InterAct the Netherlands | Quantitative questionnaire | Grams per day | Legumes | Not available | Soy products |
| EPIC-InterAct Spain | Interviewer-administered quantitative online questionnaire | Grams per day | Legumes | Not available | Not available |
| EPIC-InterAct Sweden | FFQ | Grams per day | Legumes | Not available | Not available |
| EPIC-InterAct UK | FFQ | Grams per day | Legumes | Not available | Soy products |
| FMC | Interviewer-administered dietary history | Grams per day | Dried pea  Fresh pea  Fresh green bean  Peanut | Dried pea  Fresh pea  Fresh green bean | Not available |
| Hoorn | FFQ | Grams per day | Legumes  Peanuts | Not available | Tempeh, tahoe |
| SUN | FFQ | Grams per day | Total legumes | Lentils  Peas  Chickpeas  Beans | Not available |
| Whitehall II | FFQ | Serving per day | Dried lentils, beans, peas  Baked beans  Peanuts  Tofu or soya bean curd  Textured vegetable protein | Dried lentils, beans, peas  Baked beans | Tofu or soya bean curd  Textured vegetable protein |
| Zutphen Elderly | Interviewer-administered dietary history | Grams per day | Dried white/kidney beans  Dried green peas  Dried chickpeas  Dried lentils  Raw chickpeas  Canned or glass chickpeas  White beans in tomato sauce  Cooked white/kidney beans  Cooked chickpeas  Cooked lentils  Cooked green peas  Soy products | Dried white/kidney beans  Dried green peas  Dried chickpeas  Dried lentils  Raw chickpeas  Canned or glass chickpeas  White beans in tomato sauce  Cooked white/kidney beans  Cooked chickpeas  Cooked lentils  Cooked green peas | Soy products |
| AusDiab | FFQ | Grams per day | Peas  Green beans  Bean sprouts or alfalfa sprouts  Baked beans  Other beans  Tofu | Peas  Green beans  Bean sprouts or alfalfa sprouts  Baked beans  Other beans | Tofu |
| CKB | Interviewer administered FFQ | Frequency | Soybean | Not available | Soybean |
| KoGES A&A | FFQ | Grams per day | Total legumes | Beans  Green peas | Soy products |
| KoGES CAVAS | FFQ | Grams per day | Total legumes | Beans  Green peas | Soy products |

^1^ARIC, Atherosclerosis Risk in Communities study; AusDiab, the Australian Diabetes, Obesity and Lifestyle Study; CARDIA, the Coronary Artery Risk Development in Young Adults Study; CKB, the China Kadoorie Biobank; CoLaus, the Cohorte Lausannoise; COSM, the Cohort of Swedish Men; ELSA, the English Longitudinal Study of Ageing; ELSA-Brasil, the Brazilian Longitudinal Study of Adult Health; EPIC, the European Prospective Investigation into Cancer; FMC, the Finnish Mobile Clinic Health Examination Survey; KoGES CAVAS, Korean Genome and Epidemiology Study of Cardiovascular Disease Association; KoGES A&A, Korean Genome and Epidemiology Study Ansan and Ansung; MESA, the Multi-Ethnic Study of Atherosclerosis; MTC, the Mexican Teachers Cohort; PRHHP, the Puerto Rico Heart Health Program; SMC, the Swedish Mammography Cohort; SUN, the University of Navarra Follow-up Study; WHI, the Women’s Health Initiative.

**Supplemental Table 4** Portion sizes used to study the association between legume consumption and incident type 2 diabetes in InterConnect

| Study name^1^ | Variable | Original reporting quantity | Portion assigned^2^,  g |
| --- | --- | --- | --- |
| ARIC | Beans or lentils | ½ cup | 91.3 |
|  | String or green beans | ½ cup | 62.5 |
|  | Peas or lima beans | ½ cup | 83.8 |
|  | Peanut butter | 1 tbsp | 16 |
|  | Nuts | 1 oz | 28.3 |
| CARDIA | Legumes | Not available | 83.5 |
|  | Peanuts | Not available | 28.3 |
|  | Cooked dried beans | Not available | 62.5 |
|  | Nuts and seeds | Not available | 28.3 |
| CKB | Soybean | Not available | 120 |
| ELSA | Pulses | 1 tbsp | 14 |
| MESA | Beans | Medium serving | 62.5 |
|  | Pea soup | Medium serving | 126.5 |
|  | Green beans | Medium serving | 62.5 |
|  | Refried beans | Medium serving | 119 |
|  | Soy milk | Medium serving | 120 |
|  | Stir fried tofu | Medium serving | 120 |
|  | Tofu dessert | Medium serving | 120 |
|  | Peanuts | Medium serving | 28.3 |
|  | Nuts | Medium serving | 28.3 |
| MTC | Kidney bean | 1 dish | 100 |
|  | Lentil or chickpeas | 1 dish | 100 |
|  | Peas | ½ cup | 84 |
|  | Fava beans | 1 dish | 100 |
|  | Green beans (ejotes) | ½ cup | 60 |
|  | Peanuts natural | ½ cup | 35 |
|  | Peanut | 1 bag | 35 |
|  | Nuts | ½ cup | 35 |
|  | Almonds | ½ cup | 35 |
|  | Soy milk | 1 glass | 240 |
| PRHHP | Peas or beans | ¼ cup | 42 |
| WHI | Green or English peas | ½ cup | 80 |
|  | Bean soups | 1 cup | 123.5 |
|  | Refried beans | ¾ cup | 178.5 |
|  | Green or string beans | ½ cup | 62.5 |
|  | All other beans | ¾ cup | 149.5 |
|  | Soy | Not available | 126 |
|  | Peanuts | 2 tbsp | 28.3 |
|  | Nuts and seeds | 2 tbsp | 28.3 |
| Whitehall II | Dried lentils, beans, peas | Medium serving | 90 |
|  | Baked beans | Medium serving | 106 |
|  | Peanuts | Medium serving | 50 |
|  | Tofu or soya bean curd | Medium serving | 120 |
|  | Textured vegetable protein | Medium serving | 120 |
|  | Nuts | Medium serving | 50 |

^1^ARIC, Atherosclerosis Risk in Communities study; CARDIA, the Coronary Artery Risk Development in Young Adults Study; CKB, the China Kadoorie Biobank; ELSA, the English Longitudinal Study of Ageing; MESA, the Multi-Ethnic Study of Atherosclerosis; MTC, the Mexican Teachers Cohort; PRHHP, the Puerto Rico Heart Health Program; WHI, the Women’s Health Initiative.

^2^Sourced from FoodData Central available at the United States Department of Agriculture (21).

**Supplemental Table 5** Missing covariate data for studying the association between legume consumption and incident type 2 diabetes in InterConnect^1, 2^

| Study | Age | Sex | ED | SMK | COM | PA | ALC | TEI | BMI | Fruit | Veg | Fish | Meat | SSB | Dairy | WC | Family T2D |
| --- | --- | --- | --- | --- | --- | --- | --- | --- | --- | --- | --- | --- | --- | --- | --- | --- | --- |
| ARIC |  |  |  |  |  |  |  |  |  |  |  |  |  |  |  |  |  |
| CARDIA |  |  |  |  |  |  |  |  |  |  |  |  |  |  |  |  |  |
| ELSA-Brasil |  |  |  |  |  |  |  |  |  |  |  |  |  |  |  |  |  |
| MESA |  |  |  |  |  |  |  |  |  |  |  |  |  |  |  |  | X |
| MTC |  |  |  |  |  |  |  |  |  |  |  |  |  |  |  |  |  |
| PRHHP |  |  |  |  |  |  |  |  |  |  |  |  |  |  |  | X |  |
| WHI |  |  |  |  |  |  |  |  |  |  |  |  |  |  |  |  |  |
| CoLaus |  |  |  |  |  |  |  |  |  |  |  |  |  |  |  |  |  |
| COSM/SMC |  |  |  |  |  |  |  |  |  |  |  |  |  |  |  |  |  |
| ELSA |  |  |  |  |  |  |  | X |  | X | X | X | X | X | X |  | X |
| EPIC-InterAct France |  |  |  |  |  |  |  |  |  |  |  |  |  |  |  |  |  |
| EPIC-InterAct Germany |  |  |  |  |  |  |  |  |  |  |  |  |  |  |  |  |  |
| EPIC-InterAct Italy |  |  |  |  |  |  |  |  |  |  |  |  |  |  |  |  | X |
| EPIC-InterAct the Netherlands |  |  |  |  |  |  |  |  |  |  |  |  |  |  |  |  |  |
| EPIC-InterAct Spain |  |  |  |  |  |  |  |  |  |  |  |  |  |  |  |  | X |
| EPIC-InterAct Sweden |  |  |  |  |  |  |  |  |  |  |  |  |  |  |  |  |  |
| EPIC-InterAct UK |  |  |  |  |  |  |  |  |  |  |  |  |  |  |  |  |  |
| FMC |  |  |  |  |  |  | X |  |  |  |  |  |  |  |  | X |  |
| Hoorn |  |  |  |  |  |  |  |  |  |  |  |  |  |  |  |  |  |
| SUN |  |  |  |  |  |  |  |  |  |  |  |  |  |  |  |  |  |
| Whitehall II |  |  |  |  |  |  |  |  |  |  |  |  |  |  |  |  |  |
| Zutphen Elderly |  |  |  |  |  |  |  |  |  |  |  |  |  |  |  | X |  |
| Golestan |  |  |  |  |  |  |  |  |  |  |  |  |  |  |  |  | X |
| AusDiab |  |  |  |  |  |  |  |  |  |  |  |  |  | X |  |  |  |
| CKB |  |  |  |  |  |  |  | X |  |  |  |  |  | X |  |  |  |
| KoGES A&A |  |  |  |  |  |  |  |  |  |  |  |  |  |  |  |  |  |
| KoGES CAVAS |  |  |  |  |  |  |  |  |  |  |  |  |  |  |  |  |  |

^1^X=Data not available.

^2^ALC, alcohol intake; ARIC, Atherosclerosis Risk in Communities study; AusDiab, the Australian Diabetes, Obesity and Lifestyle Study; BMI, body mass index; CARDIA, the Coronary Artery Risk Development in Young Adults Study; CKB, the China Kadoorie Biobank; CoLaus, the Cohorte Lausannoise; COM, baseline comorbidities; COSM, the Cohort of Swedish Men; ED, education level; ELSA, the English Longitudinal Study of Ageing; ELSA-Brasil, the Brazilian Longitudinal Study of Adult Health; EPIC, the European Prospective Investigation into Cancer; FMC, the Finnish Mobile Clinic Health Examination Survey; KoGES CAVAS, Korean Genome and Epidemiology Study of Cardiovascular Disease Association; KoGES A&A, Korean Genome and Epidemiology Study Ansan and Ansung; MESA, the Multi-Ethnic Study of Atherosclerosis; MTC, the Mexican Teachers Cohort; PA, physical activity; PRHHP, the Puerto Rico Heart Health Program; SMC, the Swedish Mammography Cohort; SMK, smoking status; SSB, sugar sweetened beverages; SUN, the University of Navarra Follow-up Study; TEI, total energy intake; T2D, type 2 diabetes; WC, waist circumference; WHI, the Women’s Health Initiative.

**Supplemental Table 6** Coding of covariate variables to study the association between legume consumption and incident type 2 diabetes in InterConnect^1^

| Cohort/covariate | Coding |
| --- | --- |
| *AusDiab* |  |
| Age | Continuous |
| Sex | Men; women |
| Education | Secondary; trade/vocational; tertiary |
| Smoking | Never; former; current; unknown |
| Physical Activity | Sedentary; insufficient; sufficient |
| Alcohol intake | Continuous |
| Family history of diabetes | None; mother or father; both mother and father |
| Co-morbidity | Ordinal score from 0-4 |
| Energy intake | Continuous |
| Fruit intake | Continuous |
| Vegetable intake | Continuous |
| Red and processed meat intake | Continuous |
| Sugary beverage intake | Not available |
| Dairy intake | Continuous |
| Fish intake | Continuous |
| Body mass index | Continuous |
| Waist circumference | Continuous |
| *ELSA UK* |  |
| Age | Continuous |
| Sex | Men; women |
| Education | NVQ4/NVQ5/Degree or equiv; higher ed below degree; NVQ3/GCE A Level equiv; NVQ2/GCE O Level equiv; NVQ1/CSE other grade equiv; foreign/other; no qualification |
| Smoking | Continuous |
| Physical Activity | More than once a week; once a week; one to three times a month; ardly ever, or never |
| Alcohol intake | Almost every day; 5/6 days per week; 3/4 days per week; 1/2 days per week; 1/2 per month; 1 every two months; 1/2 per year; not in last year |
| Family history of diabetes | Not available |
| Co-morbidity | Ordinal score from 0-4 |
| Energy intake | Not available |
| Fruit intake | Not available |
| Vegetable intake | Not available |
| Red and processed meat intake | Not available |
| Sugary beverage intake | Not available |
| Dairy intake | Not available |
| Fish intake | Not available |
| Body mass index | Continuous |
| Waist circumference |  |
| *Golestan* |  |
| Age | Continuous |
| Sex | Men; women |
| Education | Illiterate; < 5 yrs; 6-8 yrs; 9-12 yrs; university |
| Smoking | Never; former; current |
| Physical Activity | Irregular non-intense; regular non-intense; irregular intense |
| Alcohol intake | Never used; has used |
| Family history of diabetes | Not available |
| Co-morbidity | Ordinal score from 0-4 |
| Energy intake | Continuous |
| Fruit intake | Continuous |
| Vegetable intake | Continuous |
| Red and processed meat intake | Continuous |
| Sugary beverage intake | Continuous |
| Dairy intake | Continuous |
| Fish intake | Continuous |
| Body mass index | Continuous |
| Waist circumference | Continuous |
| *InterAct* |  |
| Age | Continuous |
| Sex | Men; women |
| Education | Primary; secondary; technical/professional; longer education |
| Smoking | Never; former; current; unknown |
| Physical Activity | Low; medium; high; very high |
| Alcohol intake | Continuous |
| Family history of diabetes | Yes; no |
| Co-morbidity | Ordinal score from 0-4 |
| Energy intake | Continuous |
| Fruit intake | Continuous |
| Vegetable intake | Continuous |
| Red and processed meat intake | Continuous |
| Sugary beverage intake | Continuous |
| Dairy intake | Continuous |
| Fish intake | Continuous |
| Body mass index | Continuous |
| Waist circumference | Continuous |
| *CKB* |  |
| Age | Continuous |
| Sex | Men; women |
| Education | None; primary; middle; high; technical college; university |
| Smoking | None; occasionally; most days; daily |
| Physical Activity | Continuous |
| Alcohol intake | Abstainers; ex-weekly drinkers; reduced-intake drinkers; occasional drinkers; and current weekly drinkers |
| Family history of diabetes | Yes; no |
| Co-morbidity | Ordinal score from 0-4 |
| Energy intake | Not available |
| Fruit intake | Daily; 4-6 days/week; 1-3 days/week; monthly; never/rarely |
| Vegetable intake | Daily; 4-6 days/week; 1-3 days/week; monthly; never/rarely |
| Red and processed meat intake | Daily; 4-6 days/week; 1-3 days/week; monthly; never/rarely |
| Sugary beverage intake | Not available |
| Dairy intake | Daily; 4-6 days/week; 1-3 days/week; monthly; never/rarely |
| Fish intake | Daily; 4-6 days/week; 1-3 days/week; monthly; never/rarely |
| Body mass index | Continuous |
| Waist circumference | Continuous |
| *WHI* |  |
| Age | Continuous |
| Sex | Women |
| Education | 11 categories |
| Smoking | Never; former; current |
| Physical Activity | Continuous |
| Alcohol intake | Continuous |
| Family history of diabetes | Yes; no |
| Co-morbidity | Ordinal score from 0-4 |
| Energy intake | Continuous |
| Fruit intake | Continuous |
| Vegetable intake | Continuous |
| Red and processed meat intake | Continuous |
| Sugary beverage intake | Continuous |
| Dairy intake | Continuous |
| Fish intake | Continuous |
| Body mass index | Continuous |
| Waist circumference | Continuous |
| *MESA* |  |
| Age | Continuous |
| Sex | Men; women |
| Education | No schooling; grades 1-8; grades 9-11; completed high school/ged; some college but no degree; technical school certificate; associate degree; bachelor’s degree; graduate, professional school |
| Smoking | Never; former; current |
| Physical Activity | Continuous |
| Alcohol intake | Continuous |
| Family history of diabetes | Not available |
| Co-morbidity | Ordinal score from 0-4 |
| Energy intake | Continuous |
| Fruit intake | Continuous |
| Vegetable intake | Continuous |
| Red and processed meat intake | Continuous |
| Sugary beverage intake | Continuous |
| Dairy intake | Continuous |
| Fish intake | Continuous |
| Body mass index | Continuous |
| Waist circumference | Continuous |
| *CARDIA* |  |
| Age | Continuous |
| Sex | Men; women |
| Education | Primary; secondary; university and beyond; none |
| Smoking | Never; former; current |
| Physical Activity | Continuous |
| Alcohol intake | Continuous |
| Family history of diabetes | Ordinal score from 0-4 for mother, father, brother, sister |
| Co-morbidity | Ordinal score from 0-4 |
| Energy intake | Continuous |
| Fruit intake | Rare or never; 1 time per month; 2-3 times per month; 1 time per week; 2 times per week; 3-4 times per week; 5-6 times per week; 1 time per day; 2+ times per day |
| Vegetable intake | Rare or never; 1 time per month; 2-3 times per month; 1 time per week; 2 times per week; 3-4 times per week; 5-6 times per week; 1 time per day; 2+ times per day |
| Red and processed meat intake | Rare or never; 1 time per month; 2-3 times per month; 1 time per week; 2 times per week; 3-4 times per week; 5-6 times per week; 1 time per day; 2+ times per day |
| Sugary beverage intake | Rare or never; 1 time per month; 2-3 times per month; 1 time per week; 2 times per week; 3-4 times per week; 5-6 times per week; 1 time per day; 2+ times per day |
| Dairy intake | Rare or never; 1 time per month; 2-3 times per month; 1 time per week; 2 times per week; 3-4 times per week; 5-6 times per week; 1 time per day; 2+ times per day |
| Fish intake | Rare or never; 1 time per month; 2-3 times per month; 1 time per week; 2 times per week; 3-4 times per week; 5-6 times per week; 1 time per day; 2+ times per day |
| Body mass index | Continuous |
| Waist circumference | Continuous |
| *ARIC* |  |
| Age | Continuous |
| Sex | Men; women |
| Education | Basic education or 0 years education; intermediate education; advanced education |
| Smoking | Never; former; current |
| Physical Activity | Continuous |
| Alcohol intake | Continuous |
| Family history of diabetes | Yes; no |
| Co-morbidity | Ordinal score from 0-4 |
| Energy intake | Continuous |
| Fruit intake | 6/day; 4-6/day; 2-3/day; 1/day; 5-6/week; 2-4/week; 1/week; 1-3/month; almost never |
| Vegetable intake | 6/day; 4-6/day; 2-3/day; 1/day; 5-6/week; 2-4/week; 1/week; 1-3/month; almost never |
| Red and processed meat intake | 6/day; 4-6/day; 2-3/day; 1/day; 5-6/week; 2-4/week; 1/week; 1-3/month; almost never |
| Sugary beverage intake | 6/day; 4-6/day; 2-3/day; 1/day; 5-6/week; 2-4/week; 1/week; 1-3/month; almost never |
| Dairy intake | 6/day; 4-6/day; 2-3/day; 1/day; 5-6/week; 2-4/week; 1/week; 1-3/month; almost never |
| Fish intake | 6/day; 4-6/day; 2-3/day; 1/day; 5-6/week; 2-4/week; 1/week; 1-3/month; almost never |
| Body mass index | Continuous |
| Waist circumference | Continuous |
| *PRHHP* |  |
| Age | Continuous |
| Sex | Men |
| Education | None; grades 1-4; grades 5-8; high school - attended; high school - graduated; university - attended; university - graduated |
| Smoking | Never; former; current |
| Physical Activity | Continuous |
| Alcohol intake | Continuous |
| Family history of diabetes | No; parents only; siblings only; parents and siblings |
| Co-morbidity | Ordinal score from 0-4 |
| Energy intake | Continuous |
| Fruit intake | Continuous |
| Vegetable intake | Continuous |
| Red and processed meat intake | Continuous |
| Sugary beverage intake | Continuous |
| Dairy intake | Continuous |
| Fish intake | Continuous |
| Body mass index | Continuous |
| Waist circumference | Not available |
| *ELSA-Brasil* |  |
| Age | Continuous |
| Sex | Men; women |
| Education | Incomplete elementary school; complete elementary school; complete secondary school; university degree |
| Smoking | Never; former; current |
| Physical Activity | Continuous |
| Alcohol intake | Continuous |
| Family history of diabetes | None; mother or father; both mother and father |
| Co-morbidity | Ordinal score from 0-4 |
| Energy intake | Continuous |
| Fruit intake | Continuous |
| Vegetable intake | Continuous |
| Red and processed meat intake | Continuous |
| Sugary beverage intake | Continuous |
| Dairy intake | Continuous |
| Fish intake | Continuous |
| Body mass index | Continuous |
| Waist circumference | Continuous |
| *KoGES* |  |
| Age | Continuous |
| Sex | Men; women |
| Education | Below elementary school; middle school; high school; college; university; more than a graduate school |
| Smoking | Never; former; current |
| Physical Activity | Continuous |
| Alcohol intake | Continuous |
| Family history of diabetes | Yes; no |
| Co-morbidity | Ordinal score from 0-4 |
| Energy intake | Continuous |
| Fruit intake | Continuous |
| Vegetable intake | Continuous |
| Red and processed meat intake | Continuous |
| Sugary beverage intake | Continuous |
| Dairy intake | Continuous |
| Fish intake | Continuous |
| Body mass index | Continuous |
| Waist circumference | Continuous |
| *MTC* |  |
| Age | Continuous |
| Sex | Women |
| Education | None; primary; special education; post-primary technician; secondary; post-secondary technician; preparatory / vocational; post-vocational technician; university; postgraduate |
| Smoking | Never; former; current |
| Physical Activity | Continuous |
| Alcohol intake | Continuous |
| Family history of diabetes | Yes; no |
| Co-morbidity | Ordinal score from 0-4 |
| Energy intake | Continuous |
| Fruit intake | Continuous |
| Vegetable intake | Continuous |
| Red and processed meat intake | Continuous |
| Sugary beverage intake | Continuous |
| Dairy intake | Continuous |
| Fish intake | Continuous |
| Body mass index | Continuous |
| Waist circumference | Continuous |
| *Zutphen* |  |
| Age | Continuous |
| Sex | Men |
| Education | Primary education; low level vocational training; extended low level vocational training; vocational training; secondary education; high level applied training; higher education |
| Smoking | Never; former; current |
| Physical Activity | Continuous |
| Alcohol intake | Continuous |
| Family history of diabetes | Yes; no |
| Co-morbidity | Ordinal score from 0-4 |
| Energy intake | Continuous |
| Fruit intake | Continuous |
| Vegetable intake | Continuous |
| Red and processed meat intake | Continuous |
| Sugary beverage intake | Continuous |
| Dairy intake | Continuous |
| Fish intake | Continuous |
| Body mass index | Continuous |
| Waist circumference | Not available |
| *CoLaus* |  |
| Age | Continuous |
| Sex | Men; women |
| Education | University education; high school; apprenticeship; mandatory education |
| Smoking | Never; former; current |
| Physical Activity | Continuous |
| Alcohol intake | Continuous |
| Family history of diabetes | Yes; no |
| Co-morbidity | Ordinal score from 0-4 |
| Energy intake | Continuous |
| Fruit intake | Continuous |
| Vegetable intake | Continuous |
| Red and processed meat intake | Continuous |
| Sugary beverage intake | Continuous |
| Dairy intake | Continuous |
| Fish intake | Continuous |
| Body mass index | Continuous |
| Waist circumference | Continuous |
| *COSM/SMC* |  |
| Age | Continuous |
| Sex | Men; women |
| Education | Up to 9 years; 10-12 years; more than 12 years |
| Smoking | Never; former; current |
| Physical Activity | Continuous |
| Alcohol intake | Continuous |
| Family history of diabetes | Ordinal score from 0-3 for mother, father, siblings |
| Co-morbidity | Ordinal score from 0-4 |
| Energy intake | Continuous |
| Fruit intake | Continuous |
| Vegetable intake | Continuous |
| Red and processed meat intake | Continuous |
| Sugary beverage intake | Continuous |
| Dairy intake | Continuous |
| Fish intake | Continuous |
| Body mass index | Continuous |
| Waist circumference | Continuous |
| *FMC* |  |
| Age | Continuous |
| Sex | Men; women |
| Education | Nine category Nordic classification |
| Smoking | Has never smoke;,ex-smoker; only pipe or cigar; less than 15 cigarettes a day; more than 15 cigarettes a day |
| Physical Activity | No or seldom; weekly; daily |
| Alcohol intake | Not available |
| Family history of diabetes | Yes; no |
| Co-morbidity | Ordinal score from 0-4 |
| Energy intake | Continuous |
| Fruit intake | Continuous |
| Vegetable intake | Continuous |
| Red and processed meat intake | Continuous |
| Sugary beverage intake | Continuous |
| Dairy intake | Continuous |
| Fish intake | Continuous |
| Body mass index | Continuous |
| Waist circumference | Not available |
| *Hoorn* |  |
| Age | Continuous |
| Sex | Men; women |
| Education | Primary education; low vocational education; intermediate secondary education; intermediate vocational education; high secondary education; high vocational education; university; different |
| Smoking | Never; former; current |
| Physical Activity | Continuous |
| Alcohol intake | Continuous |
| Family history of diabetes | None; mother or father; both mother and father |
| Co-morbidity | Ordinal score from 0-4 |
| Energy intake | Continuous |
| Fruit intake | Continuous |
| Vegetable intake | Continuous |
| Red and processed meat intake | Continuous |
| Sugary beverage intake | Continuous |
| Dairy intake | Continuous |
| Fish intake | Continuous |
| Body mass index | Continuous |
| Waist circumference | Continuous |
| *SUN* |  |
| Age | Continuous |
| Sex | Men; women |
| Education | None; doctoral thesis; degree; associate's degree; master |
| Smoking | Never; former; current |
| Physical Activity | Continuous |
| Alcohol intake | Continuous |
| Family history of diabetes | None; mother or father; both mother and father |
| Co-morbidity | Ordinal score from 0-4 |
| Energy intake | Continuous |
| Fruit intake | Continuous |
| Vegetable intake | Continuous |
| Red and processed meat intake | Continuous |
| Sugary beverage intake | Continuous |
| Dairy intake | Continuous |
| Fish intake | Continuous |
| Body mass index | Continuous |
| Waist circumference | Continuous |
| *Whitehall II* |  |
| Age | Continuous |
| Sex | Men; women |
| Education | No academic qualification; ordinary level; advanced level; BA/BSc,; higher degrees |
| Smoking | Never; former; current |
| Physical Activity | Inactive; moderate; active |
| Alcohol intake | Continuous |
| Family history of diabetes | Yes; no |
| Co-morbidity | Ordinal score from 0-4 |
| Energy intake | Continuous |
| Fruit intake | Continuous |
| Vegetable intake | Continuous |
| Red and processed meat intake | Continuous |
| Sugary beverage intake | Continuous |
| Dairy intake | Continuous |
| Fish intake | Continuous |
| Body mass index | Continuous |
| Waist circumference | Continuous |

^1^ARIC, Atherosclerosis Risk in Communities study; AusDiab, the Australian Diabetes, Obesity and Lifestyle Study; CARDIA, the Coronary Artery Risk Development in Young Adults Study; CKB, the China Kadoorie Biobank; CoLaus, the Cohorte Lausannoise; COSM, the Cohort of Swedish Men; ELSA, the English Longitudinal Study of Ageing; ELSA-Brasil, the Brazilian Longitudinal Study of Adult Health; EPIC, the European Prospective Investigation into Cancer; FMC, the Finnish Mobile Clinic Health Examination Survey; KoGES, Korean Genome and Epidemiology Study; MESA, the Multi-Ethnic Study of Atherosclerosis; MTC, the Mexican Teachers Cohort; PRHHP, the Puerto Rico Heart Health Program; SMC, the Swedish Mammography Cohort; SUN, the University of Navarra Follow-up Study; WHI, the Women’s Health Initiative.

**Supplemental Table 7** Summary incidence rate ratios and 95% confidence intervals for the association between consumption of total legumes (per 20 g/day) and clinically incident type 2 diabetes in InterConnect^1,2,3^

|  | Participants (New T2D cases) | IRR (95% CI) | *I*^2^ |
| --- | --- | --- | --- |
| Model 1 | 729,998 (34,893) | 1.02 (1.00, 1.03) | 70% |
| Model 2 | 729,998 (34,893) | 1.03 (1.01, 1.03) | 80% |
| Model 3 | 729,998 (34,893) | 1.02 (1.01, 1.04) | 79% |
| Model 4 | 729,998 (34,893) | 1.02 (1.00, 1.03) | 59% |
| Model 4 plus family history of diabetes | 729,998 (34,893) | 1.02 (1.00, 1.03) | 53% |
| Model 4 plus waist circumference | 729,998 (34,893) | 1.02 (1.00, 1.03) | 64% |

^1^Model 1 adjusted for sociodemographic characteristics and lifestyle covariates (age, sex, education, smoking, physical activity, alcohol intake, energy intake); Model 2 additionally included BMI; Model 3 was as Model 2 plus prevalent baseline comorbidities (hypertension, cancer, stroke, and myocardial infarction); Model 4 was as Model 3 plus food intakes including fruit, vegetables, fish, red and processed meat, sugary drinks and dairy products.

^2^Studies included: Atherosclerosis Risk in Communities study, the Australian Diabetes, Obesity and Lifestyle Study, the Brazilian Longitudinal Study of Adult Health, the China Kadoorie Biobank, the Cohort of Swedish Men and Swedish Mammography Cohort, the Coronary Artery Risk Development in Young Adults Study, the Finnish Mobile Clinic Health Examination Survey, the Golestan Cohort Study Korean Genome and Epidemiology Study of Cardiovascular Disease Association, the Multi-Ethnic Study of Atherosclerosis, the Puerto Rico Heart Health Program, the University of Navarra Follow-up Study, the Whitehall II Study, the Women’s Health Initiative, and the European Prospective Investigation into Cancer-InterAct study in France, Germany, Italy, Netherlands, Spain, Sweden and UK.

^3^IRR, incidence rate ratio; T2D, type 2 diabetes.

| 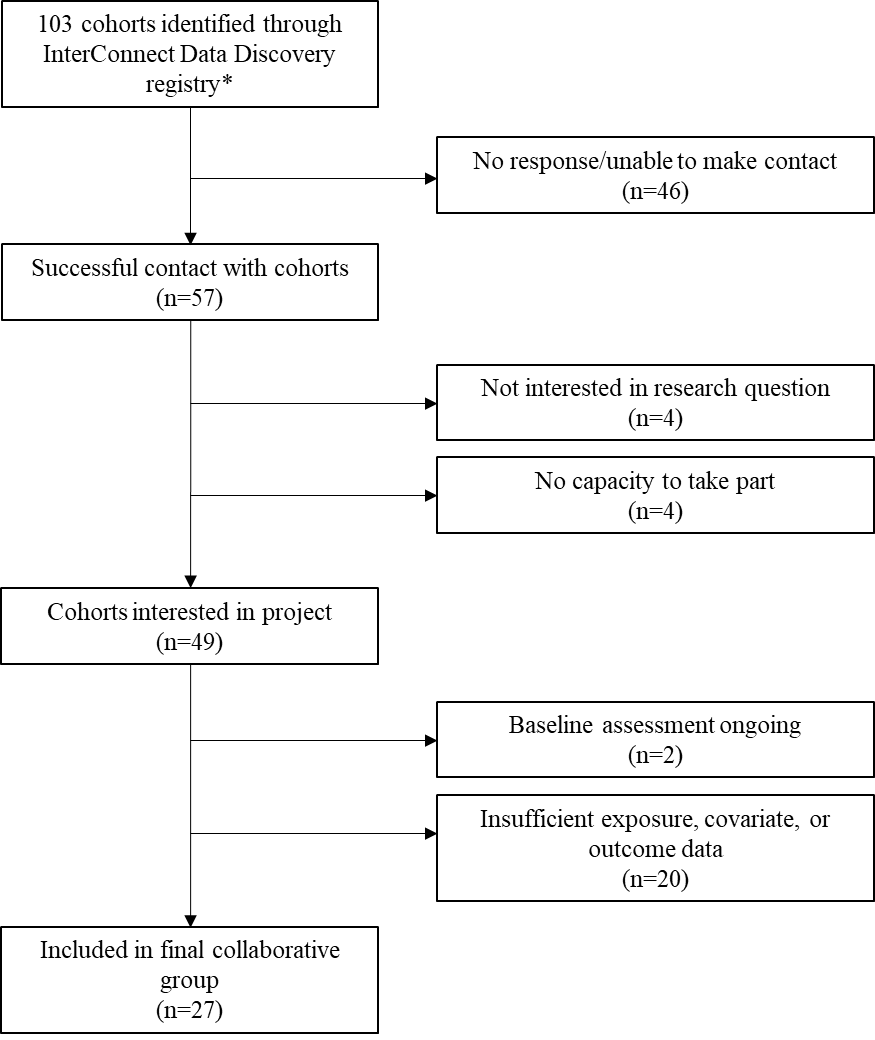 |
| --- |
| **Supplemental Figure 1** Recruitment of cohorts to study the association between legume consumption and incident type 2 diabetes in InterConnect.  *The InterConnect registry was compiled using systematic searches of the literature alongside surveys of other online study registries, surveys of websites relating to consortia of studies, and searches of the grey literature to identify unpublished data. |

| 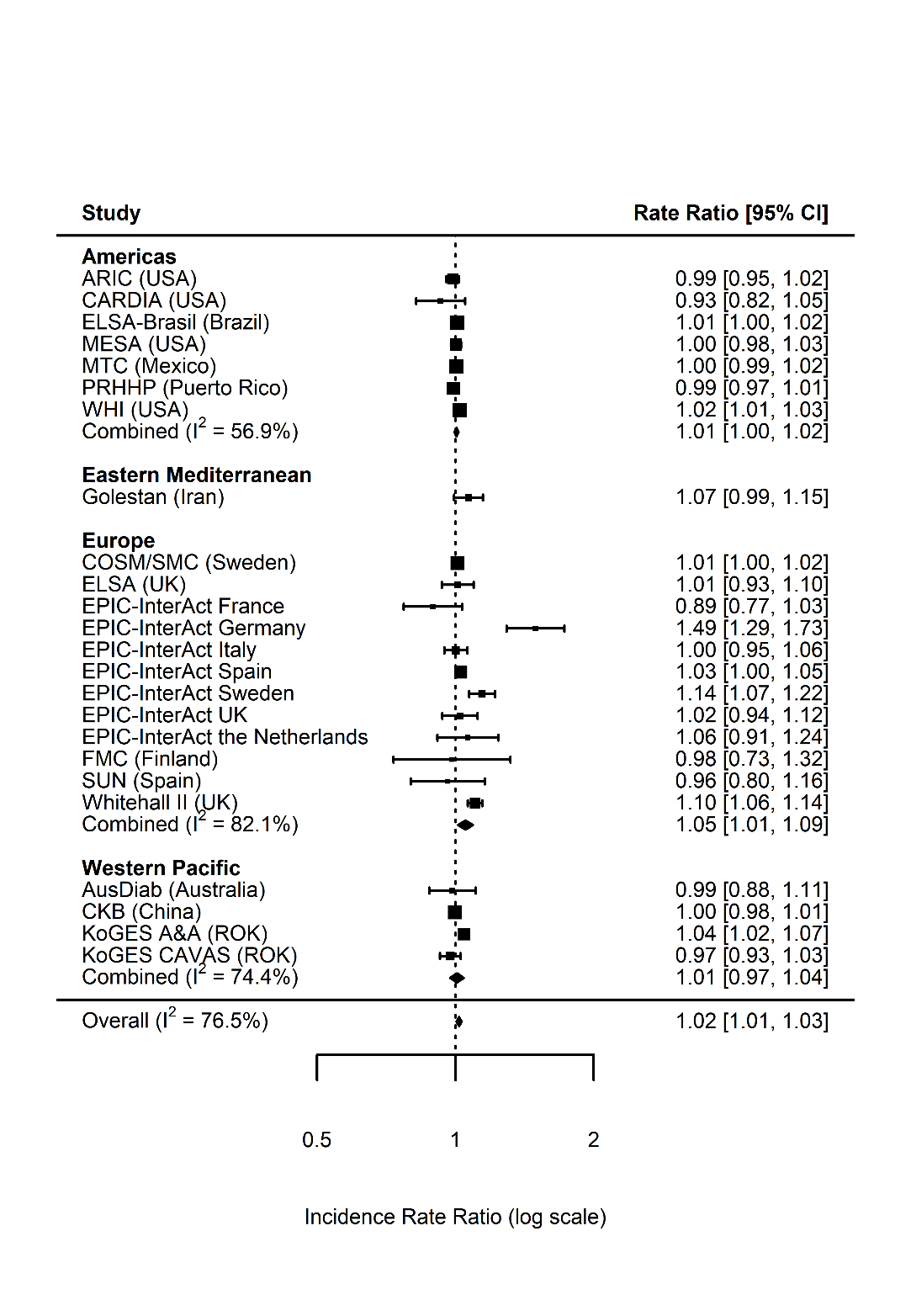 |
| --- |
| **Supplemental Figure 2** Incidence rate ratios and 95% confidence intervals for the association between the consumption of total legumes (per 20 g/day) and incident type 2 diabetes (secondary outcome) in InterConnect. Associations are adjusted for age, sex, education, smoking, physical activity, alcohol intake, total energy intake, body mass index, comorbidity (hypertension, cancer, stroke, and myocardial infarction) and other food intakes including fruit, vegetable, fish, red and processed meat, sugary drinks and dairy products.  ^1^ARIC, Atherosclerosis Risk in Communities study; AusDiab, the Australian Diabetes, Obesity and Lifestyle Study; CARDIA, the Coronary Artery Risk Development in Young Adults Study; CKB, the China Kadoorie Biobank; COSM, the Cohort of Swedish Men; ELSA, the English Longitudinal Study of Ageing; ELSA-Brasil, the Brazilian Longitudinal Study of Adult Health; EPIC, the European Prospective Investigation into Cancer; FMC, the Finnish Mobile Clinic Health Examination Survey; KoGES CAVAS, Korean Genome and Epidemiology Study of Cardiovascular Disease Association; KoGES A&A, Korean Genome and Epidemiology Study Ansan and Ansung; MESA, the Multi-Ethnic Study of Atherosclerosis; MTC, the Mexican Teachers Cohort; PRHHP, the Puerto Rico Heart Health Program; SMC, the Swedish Mammography Cohort; SUN, the University of Navarra Follow-up Study; WHI, the Women’s Health Initiative. |
| 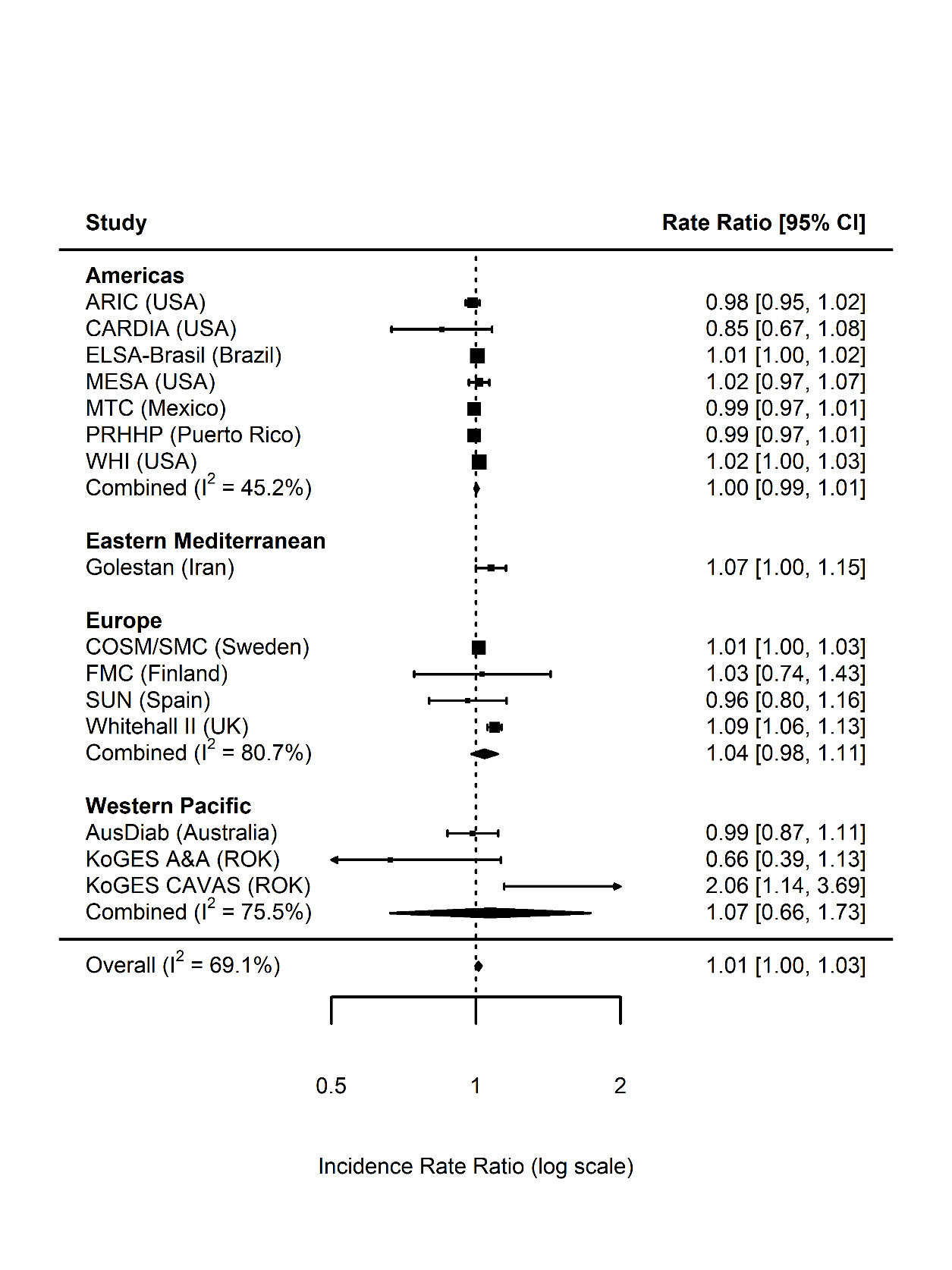 |
| **Supplemental Figure 3** Incidence rate ratios and 95% confidence intervals for the association between the consumption of pulses (per 20 g/day) and incident type 2 diabetes (secondary outcome) in InterConnect. Associations are adjusted for age, sex, education, smoking, physical activity, alcohol intake, total energy intake, body mass index, comorbidity (hypertension, cancer, stroke, and myocardial infarction) and other food intakes including fruit, vegetable, fish, red and processed meat, sugary drinks and dairy products.  ^1^ARIC, Atherosclerosis Risk in Communities study; AusDiab, the Australian Diabetes, Obesity and Lifestyle Study; CARDIA, the Coronary Artery Risk Development in Young Adults Study; COSM, the Cohort of Swedish Men; ELSA-Brasil, the Brazilian Longitudinal Study of Adult Health; FMC, the Finnish Mobile Clinic Health Examination Survey; KoGES CAVAS, Korean Genome and Epidemiology Study of Cardiovascular Disease Association; KoGES A&A, Korean Genome and Epidemiology Study Ansan and Ansung; MESA, the Multi-Ethnic Study of Atherosclerosis; MTC, the Mexican Teachers Cohort; PRHHP, the Puerto Rico Heart Health Program; SMC, the Swedish Mammography Cohort; SUN, the University of Navarra Follow-up Study; WHI, the Women’s Health Initiative. |
| 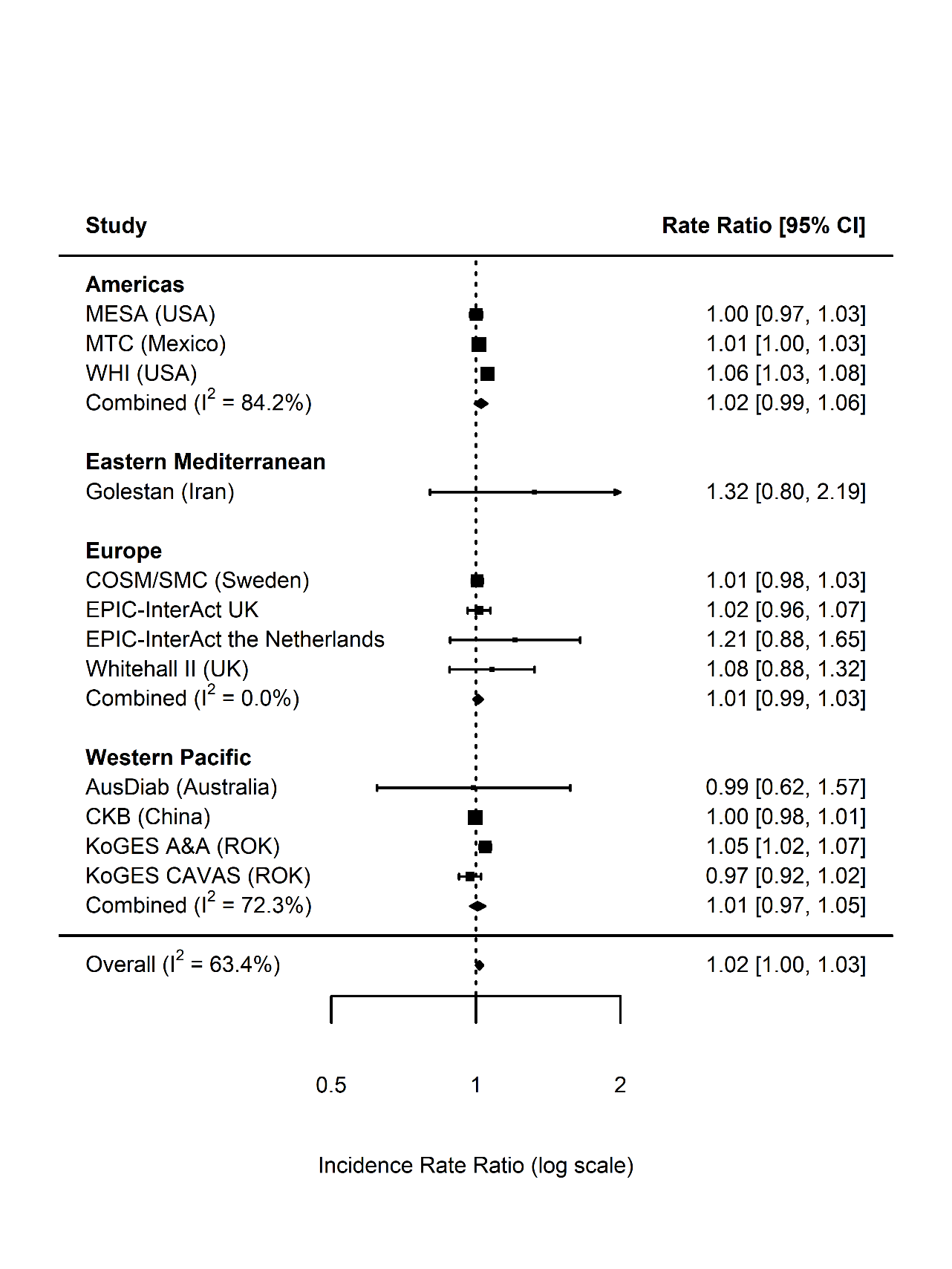 |
| **Supplemental Figure 4** Incidence rate ratios and 95% confidence intervals for the association between the consumption of soy products (per 20 g/day) and incident type 2 diabetes (secondary outcome) in InterConnect. Associations are adjusted for age, sex, education, smoking, physical activity, alcohol intake, total energy intake, body mass index, comorbidity (hypertension, cancer, stroke, and myocardial infarction) and other food intakes including fruit, vegetable, fish, red and processed meat, sugary drinks and dairy products.  ^1^AusDiab, the Australian Diabetes, Obesity and Lifestyle Study; CKB, the China Kadoorie Biobank; COSM, the Cohort of Swedish Men; EPIC, the European Prospective Investigation into Cancer; KoGES CAVAS, Korean Genome and Epidemiology Study of Cardiovascular Disease Association; KoGES A&A, Korean Genome and Epidemiology Study Ansan and Ansung; MESA, the Multi-Ethnic Study of Atherosclerosis; MTC, the Mexican Teachers Cohort; SMC, the Swedish Mammography Cohort; WHI, the Women’s Health Initiative. |
| 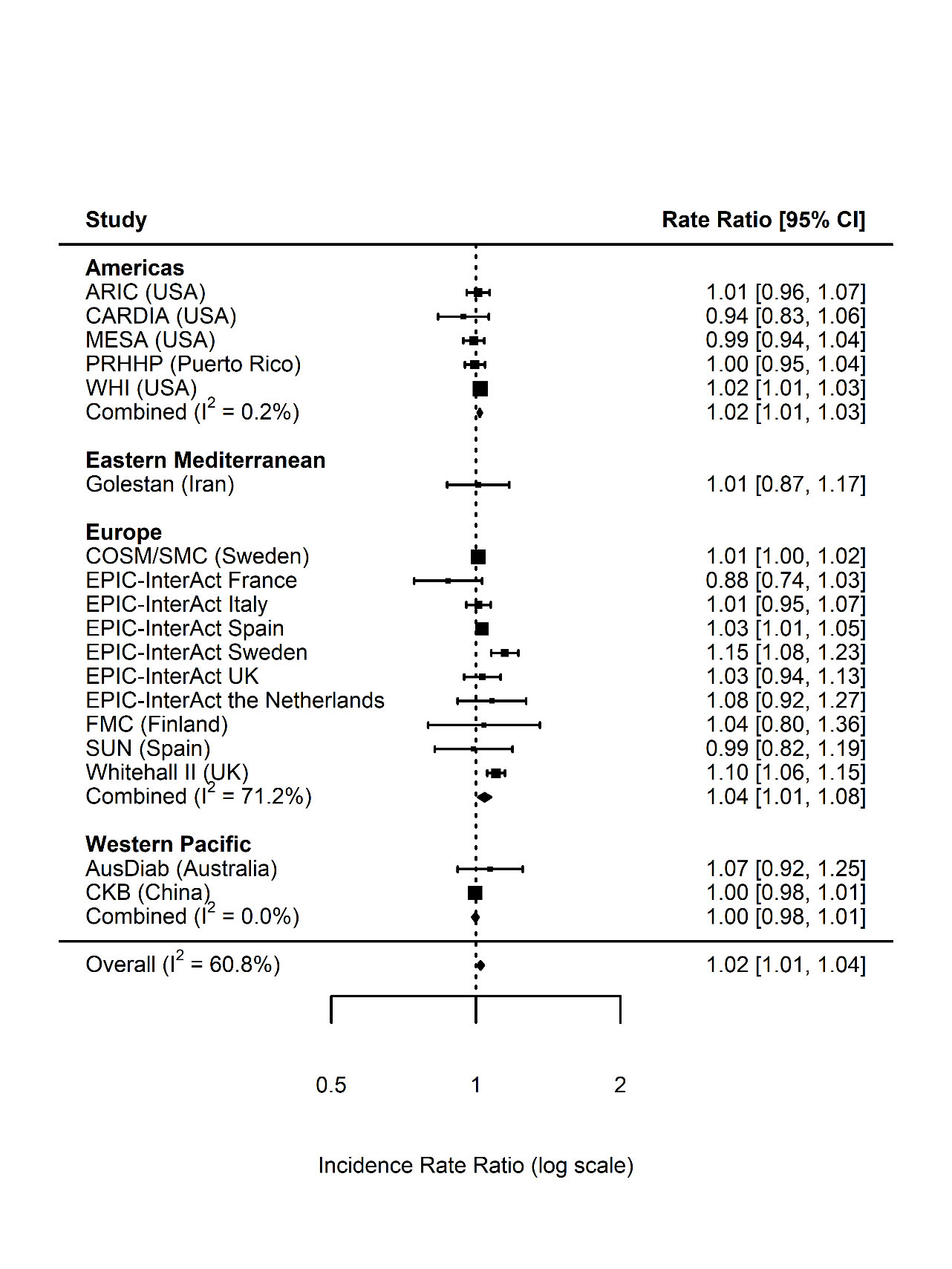 |
| **Supplemental Figure 5** Incidence rate ratios and 95% confidence intervals for the association between the consumption of total legumes (per 20 g/day) and incident type 2 diabetes (primary outcome) in InterConnect. New-onset cases of T2D in the first two years of follow-up are excluded. Associations are adjusted for age, sex, education, smoking, physical activity, alcohol intake, total energy intake, body mass index, comorbidity (hypertension, cancer, stroke, and myocardial infarction), fruit intake, vegetable intake, red and processed meat intake, sugary drinks intake, dairy intake, fish intake.  ^1^ARIC, Atherosclerosis Risk in Communities study; AusDiab, the Australian Diabetes, Obesity and Lifestyle Study; CARDIA, the Coronary Artery Risk Development in Young Adults Study; CKB, the China Kadoorie Biobank; COSM, the Cohort of Swedish Men; EPIC, the European Prospective Investigation into Cancer; FMC, the Finnish Mobile Clinic Health Examination Survey; MESA, the Multi-Ethnic Study of Atherosclerosis; PRHHP, the Puerto Rico Heart Health Program; SMC, the Swedish Mammography Cohort; SUN, the University of Navarra Follow-up Study; WHI, the Women’s Health Initiative. |
| 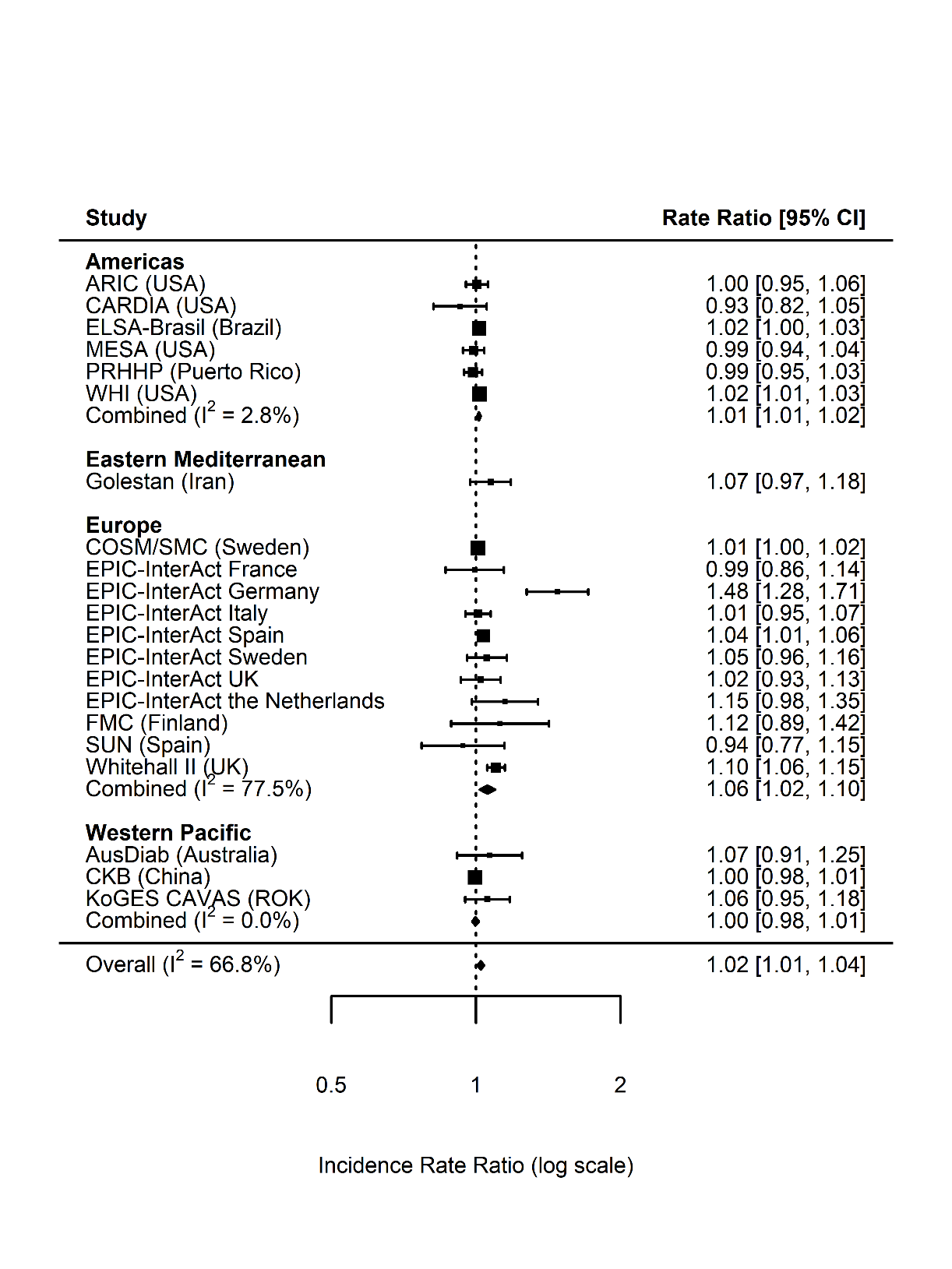 |
| **Supplemental Figure 6** Incidence rate ratios and 95% confidence intervals for the association between the consumption of total legumes (per 20 g/day) and incident type 2 diabetes (primary outcome) in InterConnect excluding non-consumers. Associations are adjusted for age, sex, education, smoking, physical activity, alcohol intake, total energy intake, body mass index, comorbidity (hypertension, cancer, stroke, and myocardial infarction), fruit intake, vegetable intake, red and processed meat intake, sugary drinks intake, dairy intake, fish intake.  ^1^ARIC, Atherosclerosis Risk in Communities study; AusDiab, the Australian Diabetes, Obesity and Lifestyle Study; CARDIA, the Coronary Artery Risk Development in Young Adults Study; CKB, the China Kadoorie Biobank; COSM, the Cohort of Swedish Men; ELSA-Brasil, the Brazilian Longitudinal Study of Adult Health; EPIC, the European Prospective Investigation into Cancer; FMC, the Finnish Mobile Clinic Health Examination Survey; KoGES CAVAS, Korean Genome and Epidemiology Study of Cardiovascular Disease Association; MESA, the Multi-Ethnic Study of Atherosclerosis; PRHHP, the Puerto Rico Heart Health Program; SMC, the Swedish Mammography Cohort; SUN, the University of Navarra Follow-up Study; WHI, the Women’s Health Initiative. |
| 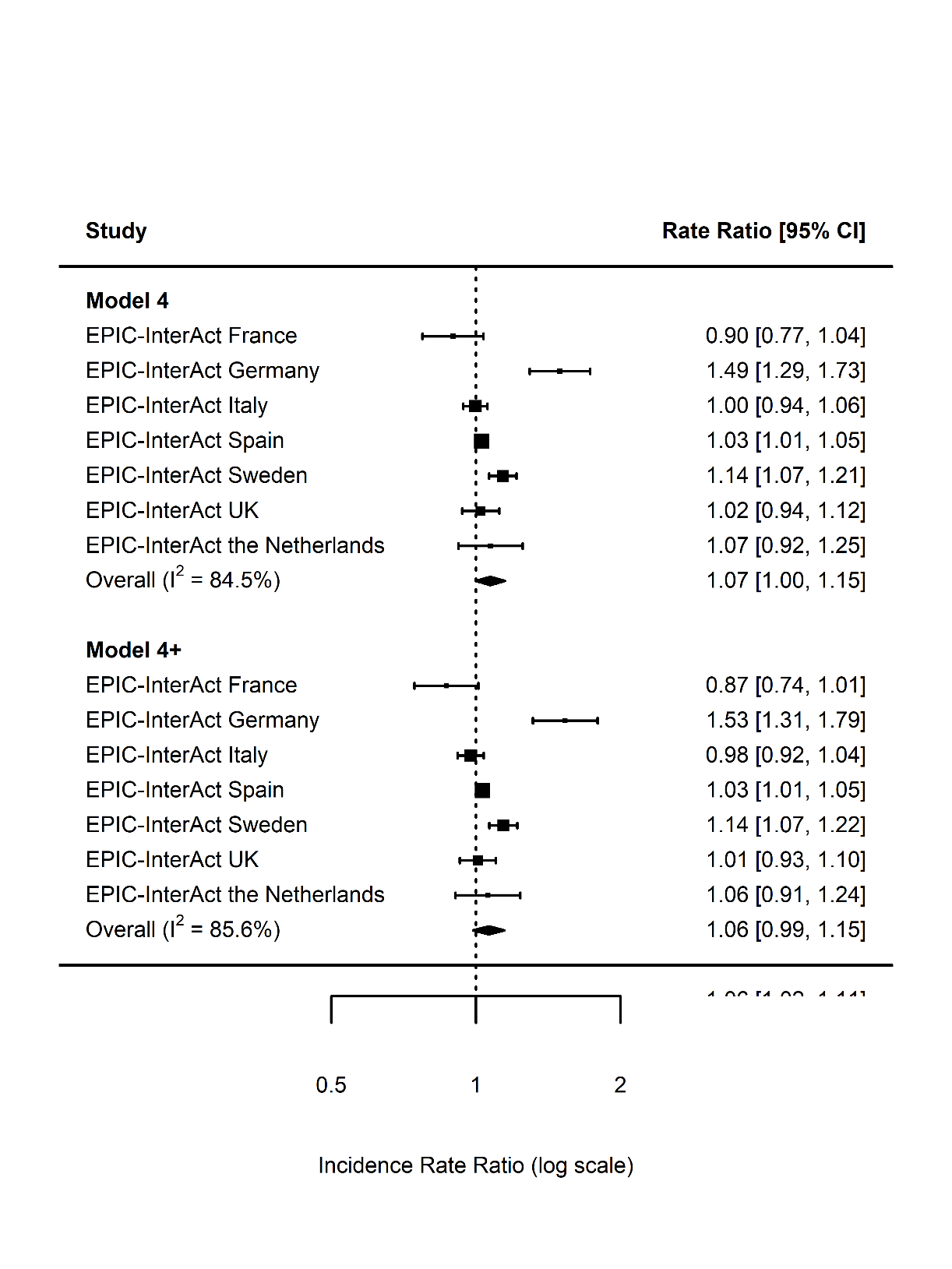 |
| **Supplemental Figure 7** Incidence rate ratios and 95% confidence intervals for the association between the consumption of total legumes (per 20 g/day) and incident type 2 diabetes (primary outcome) in EPIC-InterAct, with and without additional adjustment for tea, coffee, cereal products, eggs, potato, soups, and sugars, as well as use of hormone replacement therapy. For model 4, associations are adjusted for age, sex, education, smoking, physical activity, alcohol intake, total energy intake, body mass index, comorbidity (hypertension, cancer, stroke, and myocardial infarction), fruit intake, vegetable intake, red and processed meat intake, sugary drinks intake, dairy intake, fish intake. Model 4+ additionally adjusts for consumption of tea, coffee, cereal products, eggs, potato, soups, and sugars, as well as use of hormone replacement therapy (women only).  ^1^EPIC, The European Prospective Investigation into Cancer. |

| 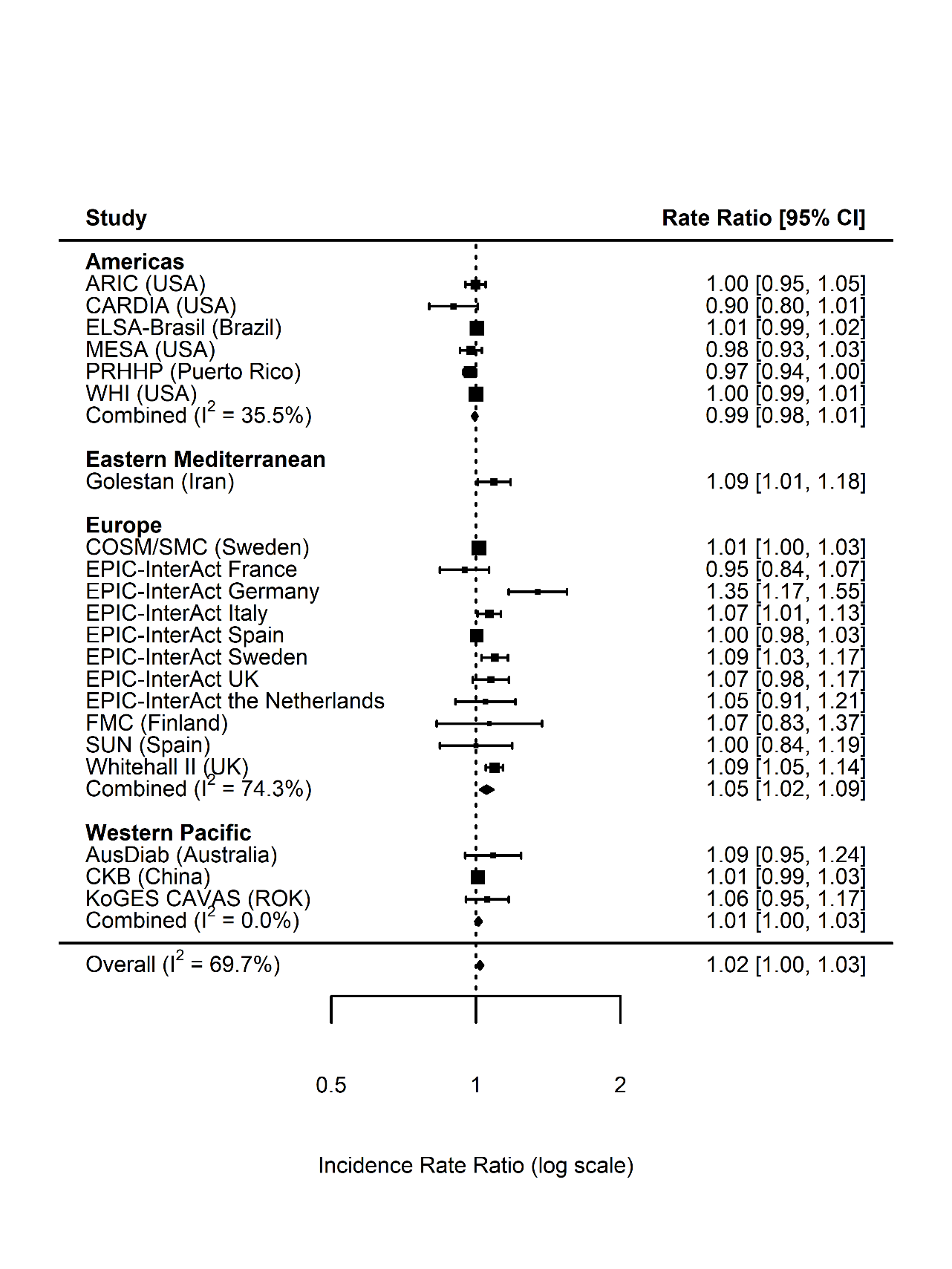 |
| --- |
| **Supplemental Figure 8** Incidence rate ratios and 95% confidence intervals for the association between the consumption of total legumes (per 20 g/day) and incident type 2 diabetes (primary outcome) in InterConnect. Associations are adjusted for age, sex, education, smoking, physical activity, alcohol and total energy intake.  ^1^ARIC, Atherosclerosis Risk in Communities study; AusDiab, the Australian Diabetes, Obesity and Lifestyle Study; CARDIA, the Coronary Artery Risk Development in Young Adults Study; CKB, the China Kadoorie Biobank; COSM, the Cohort of Swedish Men; ELSA-Brasil, the Brazilian Longitudinal Study of Adult Health; EPIC, the European Prospective Investigation into Cancer; FMC, the Finnish Mobile Clinic Health Examination Survey; KoGES CAVAS, Korean Genome and Epidemiology Study of Cardiovascular Disease Association; MESA, the Multi-Ethnic Study of Atherosclerosis; PRHHP, the Puerto Rico Heart Health Program; SMC, the Swedish Mammography Cohort; SUN, the University of Navarra Follow-up Study; WHI, the Women’s Health Initiative. |

| 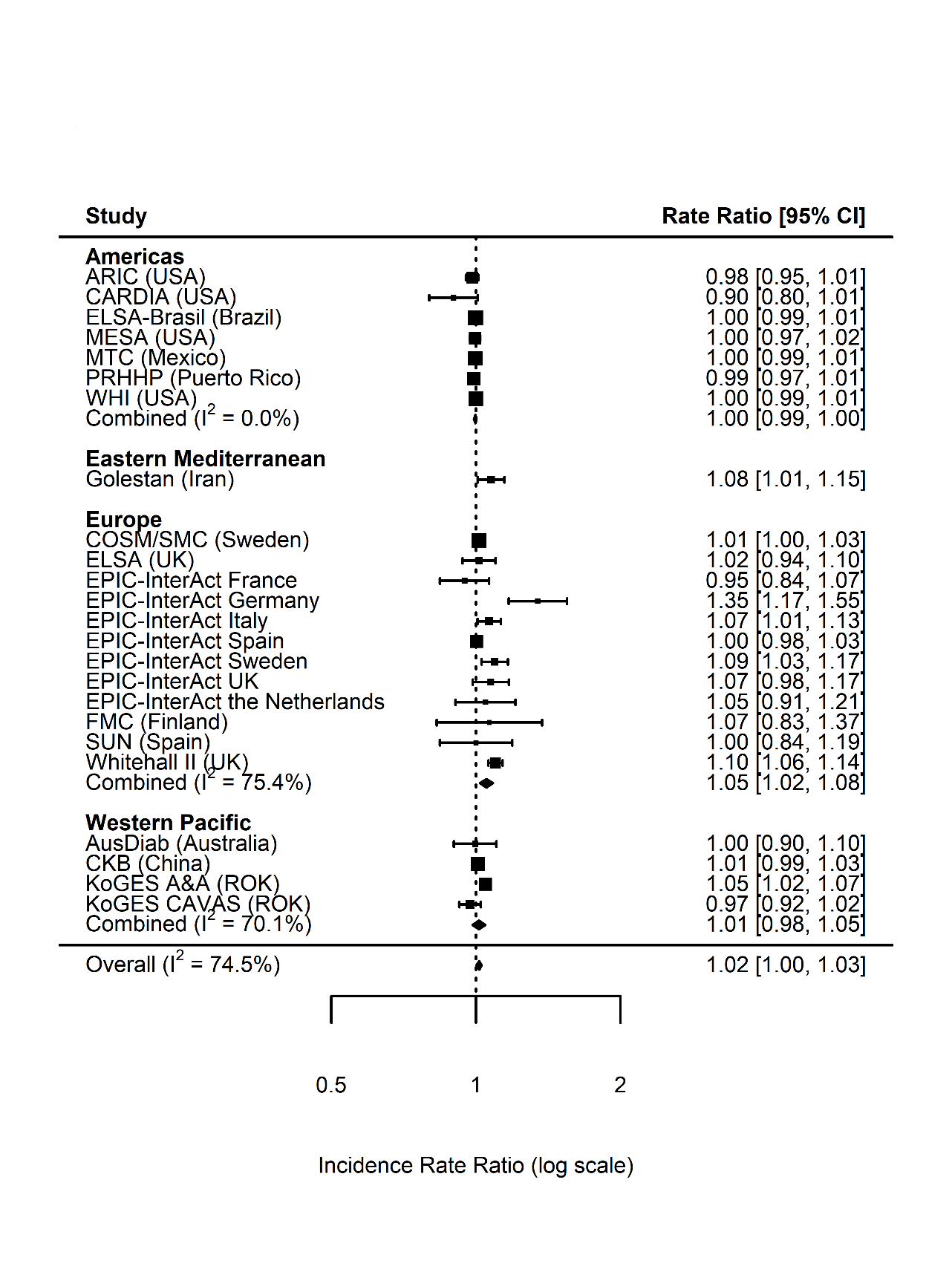 |
| --- |
| **Supplemental Figure 9** Incidence rate ratios and 95% confidence intervals for the association between the consumption of total legumes (per 20 g/day) and incident type 2 diabetes (secondary outcome) in InterConnect. Associations are adjusted for age, sex, education, smoking, physical activity, alcohol and total energy intake.  ^1^ARIC, Atherosclerosis Risk in Communities study; AusDiab, the Australian Diabetes, Obesity and Lifestyle Study; CARDIA, the Coronary Artery Risk Development in Young Adults Study; CKB, the China Kadoorie Biobank; COSM, the Cohort of Swedish Men; ELSA, the English Longitudinal Study of Ageing; ELSA-Brasil, the Brazilian Longitudinal Study of Adult Health; EPIC, the European Prospective Investigation into Cancer; FMC, the Finnish Mobile Clinic Health Examination Survey; KoGES CAVAS, Korean Genome and Epidemiology Study of Cardiovascular Disease Association; KoGES A&A, Korean Genome and Epidemiology Study Ansan and Ansung; MESA, the Multi-Ethnic Study of Atherosclerosis; MTC, the Mexican Teachers Cohort; PRHHP, the Puerto Rico Heart Health Program; SMC, the Swedish Mammography Cohort; SUN, the University of Navarra Follow-up Study; WHI, the Women’s Health Initiative. |

**Supplemental References**

1. The Atherosclerosis Risk in Communities (ARIC) Study: design and objectives. The ARIC investigators. Am J Epidemiol. 1989;129:687–702.

2. Friedman GD, Cutter GR, Donahue RP, Hughes GH, Hulley SB, Jacobs DRJ, Liu K, Savage PJ. CARDIA: study design, recruitment, and some characteristics of the examined subjects. J Clin Epidemiol. 1988;41:1105–16.

3. Aquino EML, Barreto SM, Bensenor IM, Carvalho MS, Chor D, Duncan BB, Lotufo PA, Mill JG, Molina MDC, Mota ELA, et al. Brazilian Longitudinal Study of Adult Health (ELSA-Brasil): objectives and design. Am J Epidemiol. 2012;175:315–24.

4. Nettleton JA, Steffen LM, Ni H, Liu K, Jacobs Jr. DR. Dietary patterns and risk of incident type 2 diabetes in the Multi-Ethnic Study of Atherosclerosis (MESA). Diabetes Care. 2008;31:1777–82.

5. Lajous M, Ortiz-Panozo E, Monge A, Santoyo-Vistrain R, García-Anaya A, Yunes-Díaz E, Rice MS, Blanco M, Hernández-Ávila M, Willett WC, et al. Cohort Profile: The Mexican Teachers’ Cohort (MTC). Int J Epidemiol. 2015;46:e10–e10.

6. Garcia-Palmieri MR, Sorlie P, Tillotson J, Costas RJ, Cordero E, Rodriguez M. Relationship of dietary intake to subsequent coronary heart disease incidence: The Puerto Rico Heart Health Program. Am J Clin Nutr. 1980;33:1818–27.

7. Design of the Women’s Health Initiative clinical trial and observational study. The Women’s Health Initiative Study Group. Control Clin Trials. 1998;19:61–109.

8. Pourshams A, Khademi H, Malekshah AF, Islami F, Nouraei M, Sadjadi AR, Jafari E, Rakhshani N, Salahi R, Semnani S, et al. Cohort Profile: The Golestan Cohort Study--a prospective study of oesophageal cancer in northern Iran. Int J Epidemiol. 2010;39:52–9.

9. Firmann M, Mayor V, Vidal PM, Bochud M, Pécoud A, Hayoz D, Paccaud F, Preisig M, Song KS, Yuan X, et al. The CoLaus study: a population-based study to investigate the epidemiology and genetic determinants of cardiovascular risk factors and metabolic syndrome. BMC Cardiovasc Disor. 2008;8:6.

10. Harris H, Håkansson N, Olofsson C, Julin B, Åkesson A WA. The Swedish mammography cohort and the cohort of Swedish men: Study design and characteristics of 2 population-based longitudinal cohorts. OA Epidemiol. 2013;1.

11. Steptoe A, Breeze E, Banks J, Nazroo J. Cohort profile: the English longitudinal study of ageing. Int J Epidemiol. 2013;42:1640–8. Available from:

12. Forouhi NG, Wareham NJ. The EPIC-InterAct Study: A Study of the Interplay between Genetic and Lifestyle Behavioral Factors on the Risk of Type 2 Diabetes in European Populations. Curr Nutr Rep. 2014;3:355–63.

13. Knekt P, Rissanen H, Jarvinen R, Heliovaara M. Cohort Profile: The Finnish Mobile Clinic Health Surveys FMC, FMCF and MFS. Int J Epidemiol. 2017;46:1760-1761i.

14. Rutters F, Nijpels G, Elders P, Stehouwer CDA, van der Heijden AA, Groeneveld L, ‘T Hart LM, Dekker JM, Beulens JWJ. Cohort Profile: The Hoorn Studies. Int J Epidemiol. 2017;47:396-396j.

15. Martinez-Gonzalez MA. The SUN cohort study (Seguimiento University of Navarra). Public Heal Nutr. 2006;9:127–31.

16. Marmot M, Brunner E. Cohort Profile: The Whitehall II study. Int J Epidemiol. 2005;34:251–6.

17. Dower JI, Geleijnse JM, Hollman PCH, Soedamah-Muthu SS, Kromhout D. Dietary epicatechin intake and 25-y risk of cardiovascular mortality: the Zutphen Elderly Study. Am J Clin Nutr. 2016;104:58–64.

18. Dunstan DW, Zimmet PZ, Welborn TA, Cameron AJ, Shaw J, de Courten M, Jolley D, McCarty DJ. The Australian Diabetes, Obesity and Lifestyle Study (AusDiab)--methods and response rates. Diabetes Res Clin Pr. 2002;57:119–29.

19. Chen Z, Chen J, Collins R, Guo Y, Peto R, Wu F, Li L, Group CKB (CKB) collaborative. China Kadoorie Biobank of 0.5 million people: survey methods, baseline characteristics and long-term follow-up. Int J Epidemiol. 2011;40:1652–66.

20. Kim Y, Han B-G, Group K. Cohort Profile: The Korean Genome and Epidemiology Study (KoGES) Consortium. Int J Epidemiol. 2017;46:e20–e20.

21. U.S. Department of Agriculture. FoodData Central [Internet]. 2020. Available from: https://fdc.nal.usda.gov/
